# Understanding Acceptable Level of Risk: Incorporating the Economic Cost of Under-Managing Invasive Species

**DOI:** 10.1371/journal.pone.0141958

**Published:** 2015-11-04

**Authors:** Alisha D. Davidson, Chad L. Hewitt, Donna R. Kashian

**Affiliations:** 1 Wayne State University, 5047 Gullen Mall, Detroit, MI, 48202, United States of America; 2 University of Waikato, Hamilton, 3240, New Zealand; Central Michigan University, UNITED STATES

## Abstract

Management of nonindigenous species includes prevention, early detection and rapid response and control. Early detection and rapid response depend on prioritizing and monitoring sites at risk for arrival or secondary spread of nonindigenous species. Such monitoring efforts require sufficient biosecurity budgets to be effective and meet management or policy directives for reduced risk of introduction. Such consideration of risk reduction is rarely considered, however. Here, we review the concepts of acceptable level of risk (ALOR) and associated costs with respect to nonindigenous species and present a framework for aligning risk reduction priorities with available biosecurity resources. We conclude that available biosecurity resources may be insufficient to attain stated and desired risk reduction. This outcome highlights the need to consider policy and management directives when beginning a biosecurity program to determine the feasibility of risk reduction goals, given available resources.

## Introduction

The wide-ranging impacts of nonindigenous species have spurred development of a suite of management efforts that can be applied at various stages of the invasion process e.g., [1,2]. These management efforts are often divided into three components: prevention, early detection and rapid response (EDRR) and control [3]. Prevention primarily consists of limiting the arrival of new populations or species and may include limiting the strength of the vector by decreasing either the number, the diversity, or the quality of propagules in the vector. Early detection and rapid response consists of detecting and eradicating new populations. Control consists of monitoring and reducing or ameliorating the impacts of existing populations. These monitoring efforts often consist of identifying sites at greatest risk of arrival or secondary spread or those areas currently most impacted by populations of nonindigenous species, and identifying optimal detection methods for taxa of concern e.g., [4,5]. The methods used to determine investment strategies for biosecurity decision-making typically include risk assessment and cost-benefit analysis that compare costs of action in relation to costs associated with inaction e.g., [6]). For example, Haight and Polasky [7] explored how management decisions change with certainty regarding the level of infestation. They found that no action is warranted with a large probability of no infestation. Sims and Finnoff [8] also looked at management action related to knowledge of the spread in a political context and found that a species that spreads in an unpredictable manner warrants immediate action, while a slow-spreading species may allow a delayed response.

Risk assessment can be used at each stage to prioritize vectors and sites for monitoring or control efforts. Continued development of programs within each of the three components occurs from international [9] to local [10] levels. Design and implementation of these programs often involve a compromise between available resources and achievable aims [6,8, 11,12]. Ballast water management is an example of this compromise.

Ballast water exchange (BWE), the replacement of coastal water with open-ocean water during navigation, has been the primary approach to limiting this vector. Yet efficiency of BWE in removing organisms, while often greater than 80%, can be as low as 50% [13]. Attempts to develop discharge standards that would more consistently reduce the likelihood of introductions were controversial given the uncertainty around the number of living organisms that constituted a “safe” discharge [14] and the significant costs of doing so [15]. A compromise was reached in setting very low, but not zero, discharge standards [14].

This compromise between acceptable risk and logistical constraints is reflected in the concept of acceptable level of risk (ALOR). Acceptable level of risk is used in a variety of fields, including environmental management, food safety and biomedical research e.g., [16,17,18]. This policy tool represents the highest level of risk that an entity is willing to accept, and is frequently set relative to the costs of implementing a risk-reduction policy. Decisions surrounding acceptable risk are most often informal and inexplicit. For example, individuals consider the level of (financial) risk they are willing to accept when taking out a homeowner’s policy; an individual with a higher acceptable level of risk would potentially buy a less expensive policy that provides fewer benefits in event of a disaster and an individual with a lower acceptable level of risk might buy a more expensive policy that provides greater benefits in event of a disaster.

An example of an explicit application of the ALOR concept is seen in Australia’s biosecurity policy. In an extensive review of their biosecurity strategy, Australian policy states that an ALOR of zero is unattainable and unaffordable [19]. To achieve zero risk, the movement of animals, plants, people and cargo would not be possible, resulting in a limitation of Australia’s economy and standing in violation of World Trade Organization (WTO) Sanitary and Phytosanitary (SPS) Agreement principles. As such, the Australian government set the ALOR to very low, but not to zero [20].

### A framework for integrating the ALOR into program design

Despite the widespread use of ALOR in policy, integrating ALOR into the design of management programs can be challenging [21]. For example, what is very low risk and how is it measured? How does one decide what the compromise should be between resource allocation (biosecurity costs) and achievable goals (acceptable risk)? In the past, design of management programs has typically been based around the available resources determined under budget process (biosecurity investment) which sets boundaries on operational (non-emergency) expenditure. This may result in the need to prioritise expenditure based on affordability rather than need. Hence the process would, for example, determine a suite of high(er) risk species and development of various management actions articulating costs and likely outcomes. These species would then be prioritised for action based on cost:benefit ratios within the constraints of the budget allocation. If the total costs exceed available resources, fewer species would be selected and the process repeated until an affordable program could be implemented. The outcomes of the program will therefore be dependent on projecting an appropriate budget. Whether the outcomes of the program aligned with the ALOR become a post-hoc consideration.

This approach relegates consideration of ALOR to the end of the process, or nowhere at all. Tied to the fate of ALOR in biosecurity decision-making is the acceptable rate of Type II errors. In a statistical context, Type II errors are the probability of incorrectly accepting a false null hypothesis, represented by β and denoted here as E_II_. Type II errors are the quantitative equivalent of ALOR—both represent the acceptable “miss rate” that a species of concern will not be intercepted. In contrast, Type I errors are the probability of incorrectly rejecting a true null hypothesis, represented by α and denoted here as E_I_.

For the proposed framework and associated case study, the statistical definition of Type I and II errors are extended into a management context. That is, a Type I error represents over-management of a particular entity (e.g., incorrectly assigning a high conservation status to a species or habitat), and Type II error represents under-management of a particular entity (e.g., incorrectly assigning a low conservation status to a species or habitat). The costs of these management-based Type I and Type II errors are the costs of over-management (Type I costs) and the costs of damage from under-management (Type II costs).

Costs of under-management (Type II errors) can be very high, particularly in areas of conservation management or when measured over long time frames [22]. This is because Type II errors often incur potentially irreversible environmental costs (e.g., most aquatic nonindigenous species are never eradicated), in addition to on-going management costs. Nearly 80% of surveyed aquatic biosecurity researchers and managers identified the avoidance of Type II errors as more important than avoiding Type I errors [23]. Yet scientists manage Type I errors (falsely attributing blame) through assiduous application of Null Hypothesis Significance Testing where stringent acceptance criteria are determined using an α value (typically set at 0.05).

**Fig 1:**
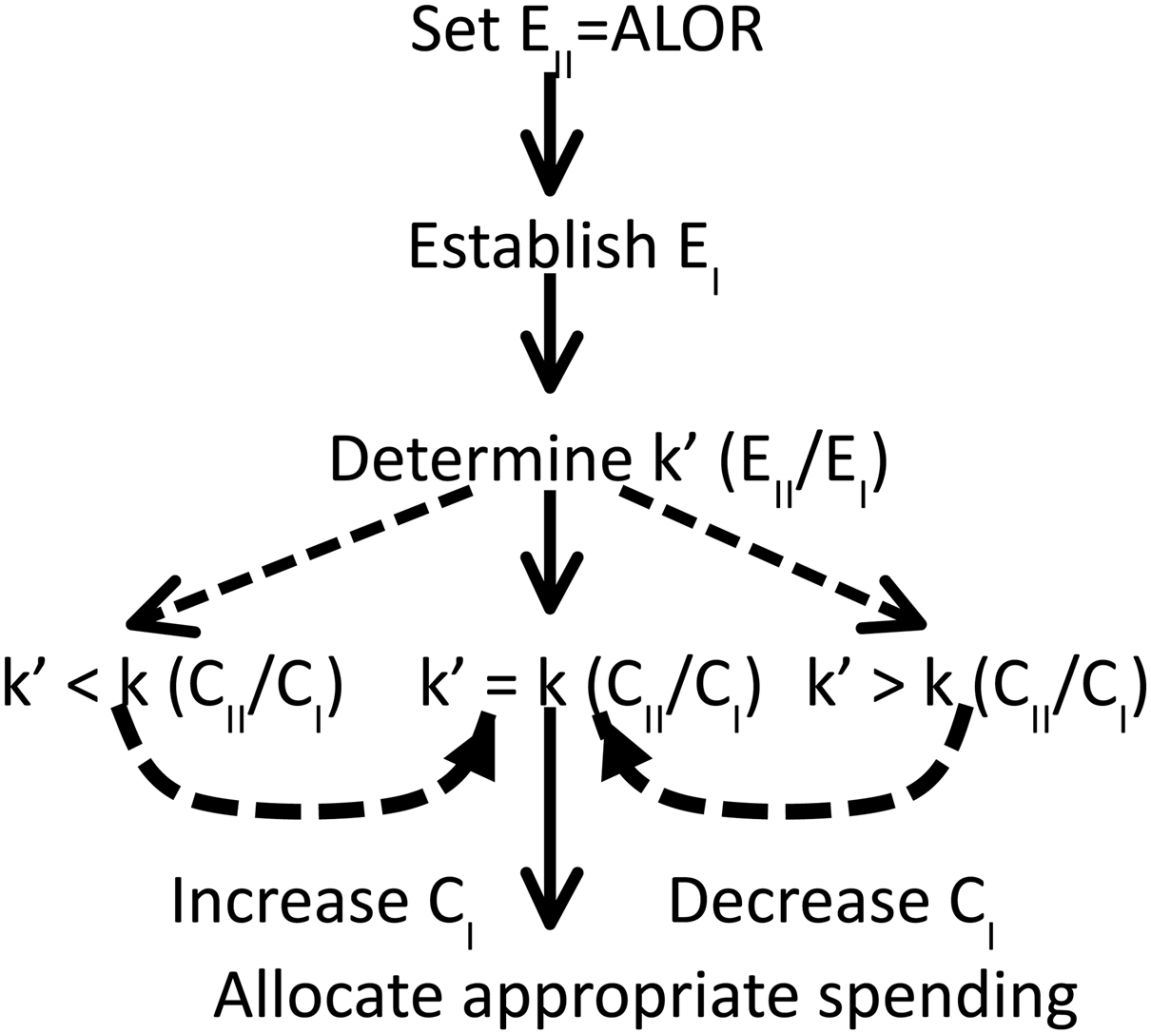
Proposed decision-support framework to incorporate Type II errors and ALOR early in program design. k’ = ratio between the acceptable Type I error rate (E_I_) and acceptable Type II error rate (E_II_); and k = ratio between costs of Type II (C_II_) and Type I (C_I_) errors.

Bringing consideration of under-management (Type II errors) alongside over-management (Type I errors) is therefore crucial to designing programs that reflect policy decisions regarding risk and that account for the impact costs of introductions. Such *a priori* consideration has been suggested by several authors [24, 25, 26]. We present a framework that moves consideration of ALOR and Type II errors to the forefront of program design and indeed provides a mechanism to consider ALOR in advising budget considerations. It proposes to use the relative costs of each error type to determine biosecurity spending that reflects the cost of species introductions (Fig 1). Given the argument that the respective error rates should reflect their respective costs [25], the ratio of acceptable Type II to Type I error rates (k’ = E_II_/E_I_) is compared to the ratio of the costs of each error type (k = C_II_/C_I_). A demonstration of how this framework can be used to aid biosecurity decision-making is presented below.

### Applying this framework to monitoring Michigan lakes

Effective eradication or control is most successful when population density and abundance are low [1,27]. Therefore, early detection and rapid response (EDRR) has become an increasingly important management tool that provides another option when prevention has failed. Early detection and rapid response may allow for a more flexible response based on the nature and extent of the introduction [4]. Indeed, the thresholds for management action (e.g. eradication, control, or no response) can be identified in advance, thus reducing the reactive nature of “rapid response” decision making. Monitoring programs designed to detect populations in the early stages of establishment are a major component of EDRR. However, there are uncertainties as to how much effort to apply.

Here, we use monitoring in an EDRR context to demonstrate the decision-support framework. Specifically, we use a pilot macrophyte survey program for inland lakes by the Michigan Department of Environmental Quality (MI DEQ) Water Resources Division. The survey protocol includes a visual inspection around the lake perimeter, snorkel search and rake toss. We integrate the MI DEQ monitoring program with results from an assessment of Michigan water bodies at risk of introduction via the recreational boating vector to apply the decision-support framework for two scenarios [28]. Scenario 1 (“current budget scenario”) determines the ratio of acceptable Type I and II error rates achieved by a standard EDRR program, using the MI DEQ pilot program as a case study. Scenario 2 (“ALOR budget scenario”) uses the ALOR indicated by a Great Lakes policy document regarding nonindigenous species (2012 Great Lake Restoration Initiative Action Plan) to determine the optimal biosecurity budget (S1 Supplementary Information), also using the MI DEQ pilot program as a case study.

## Methods

We developed parameter values based on sources related to monitoring for EDRR efforts in the Great Lakes (summarized in Table 1). The acceptable Type I error rate represents the acceptable rate of assigning a lake as high risk when it is in fact moderate or low risk (over-management). It was based on conventional values for acceptable rates of false positives, 0.05 [29,30]. The acceptable Type II error rate (equivalent to β) represents the acceptable rate of missing an introduction (under-management), and reflects the ALOR. For the ALOR budget scenario, we looked to the 2012 Great Lake Restoration Initiative Action Plan (a United States federal interagency action plan that provides funding to address environmental issues in the Great Lakes region) Long Term Goal, which states a ‘zero tolerance policy’ toward aquatic invasive species [31]. However, a zero Type II error rate is not logistically feasible (every lake would have to be surveyed), therefore here it was set to the near-zero value of 0.01. With an acceptable Type I error rate of 0.05, this represents a five-fold greater willingness to unnecessarily monitor a lake (over manage; Type I error) as to allow a species to establish (under manage; Type II error).

**Table 1:** Type I and II errors and costs for the monitoring for EDRR case study. **Variables to be determined are *x***_***1***_ **for Scenario 1 and *x***_***2***_ **for Scenario 2**.

	ErrorI	Error_II_ (ALOR)	Cost_I_	Cost_II_
Definition	Probability of zero-risk lakes surveyed	Probability of at-risk lakes not surveyed	Cost of monitoring program	Cost of species introduction
Value	0.05	Scenario 1: x_1_Scenario 2: 0.01	Scenario 1: US$64,137Scenario 2: x_2_	US$64,615,600

Type I and II costs reflect the respective costs of over- and under-management, respectively. Type I costs represent the costs of monitoring. For the current budget scenario, it was based on the MI DEQ monitoring budget for macrophytes (US$64,137/annum) (S2 Supplementary Information). Type II costs represent the costs of a species introduction. We found two cost types that could be applied to Michigan lakes: property value decline and the cost of controlling introductions. Changes in property values following changes in environmental amenities are often estimated using hedonic methods, which estimate a change in value based on an individual’s willingness to pay for a given amenity [32]. Using hedonic methods, Horsch and Lewis [33] found that property values declined by 13% following the introduction of *Myriophyllum spicatum* L. (Eurasian watermilfoil) in Wisconsin lakes. This finding of a 13% decline is applicable to this case study for several reasons. First, while the MI DEQ program targets macrophytes other than Eurasian watermilfoil, nuisance macrophytes generally have similar impacts (e.g., form dense submerged or floating mats that hinder recreation) [34]). Second, the culture of recreational lake use between Wisconsin and Michigan is similar, indicating the decline in property values would also be very similar [35]. To determine the cost of species introductions related to property value decline, waterfront properties sold over a three-month period (from March 9-June 9, 2014) were gathered from the Southwestern Michigan Regional Information Center real estate database (S3 Supplementary Information), which captured properties from the western half of Michigan’s Lower Peninsula. To remove the influences of several extremely high-value outliers, median values were calculated. Analysis of 337 property values gave a median value of US$225,000/property. With the 13% figures from Horsch and Lewis [33], this yielded a median decrease in property value of US$29,250/property. A conservative estimate of one property sold per lake per year was assumed.

The cost of species introductions related to control efforts was determined by contacting twelve Michigan lake groups that fund control of aquatic macrophytes through improvement boards, special assessment districts or donations (S4 SupplementaryInformation ; S1 Map). Lakes were selected over a range of locations, sizes, and levels of infestation. The most recent annual budgets (from 2012, 2013 or 2014) for aquatic macrophyte control from each lake were averaged across all lakes. The average cost of plant control was US$102,350/water body. Thus, total Type II costs were estimated at US$131,600/water body. This was multiplied by the number of lakes with unacceptable risk levels (i.e. greater than very low; discussed below).

Analyses of at-risk lakes were based on a dataset that gathered locations of boater trips and associated boater behaviours in Michigan [28]. This dataset was used to assign a categorical score to each water body receiving boaters. To determine this score, we weighted each trip according to whether the boater identified high-risk behaviours associated with high likelihood of transfer [36]; higher scores represent higher likelihood. For example, not removing visible mud, plants or animals from boats and trailers; not removing visible mud, plants or animals from personal equipment; not emptying all water; not allowing boats and equipment to dry at least five days before next use; not cleaning with either hot water, vinegar or salt solution, bleach solution or high pressure washing led to a higher score. For each water body, we summed all weighted trips across all boaters to determine an overall score [28]. Risk thresholds were assigned based on equal-interval categories from the maximum water body score (Table 2).

**Table 2:** Risk thresholds for water bodies included in analysis, based on mean water body score (calculated from high-risk boater behaviours).

Category	Water body score	Water bodies
Very high	25.6–32	1
High	19.2–<25.6	8
Moderate	12.8–<19.2	23
Low	6.4–<12.8	134
Very low	0–<6.4	225

Of 391 locations in Michigan captured by the survey, 166 (42%) were categorised as “low”, “moderate”, “high” or “very high” risk scores for incoming trips and had some risk of species introduction. We applied this proportion to the 1157 Michigan lakes larger than 100 acres (a size that makes them reasonably subject to boat traffic and therefore subject to introduction risk). This suggests 491 lakes (water bodies) in Michigan are exposed to the risk of introduction. Thus, the total Type II costs used in the case study was US$64,615,600 (US$131,600/water body * 491 water bodies).

The parameters used for the current budget scenario (Scenario 1) were Type II and Type I costs. From these, we determined the ratio of acceptable Type II to Type I error rates, k’, suggested by the cost ratio, k. The parameters used for the ALOR budget scenario (Scenario 2) were the ratio of Type II to Type I error rates and Type II costs. From these, we determine the Type I costs suggested by the desired ALOR by harmonizing the k and k’ ratios (from Fig 1).

## Results and Discussion

For the current budget scenario (Scenario 1), k (Cost_II_:Cost_I_, US$64,615,600/US$64,137), was 1007:1. This implies a willingness to under manage (accept Type II errors) 1007 times more often than over manage (Type I errors), and, by extension a very high ALOR (a Type II error probability threshold, β, of nearly 1). For the ALOR budget scenario (Scenario 2), the Type I costs suggested by a very low ALOR was US$323,078,000 (Cost_I_ = Cost_II_/k’ = US$64,615,600/0.2, where k’ = Error_II_/Error_I_ = 0.01/0.05). These outcomes demonstrate an enormous discrepancy between the desired (in this case mandated) level of protection and the resources currently available to achieve this protection.

**Fig 2:**
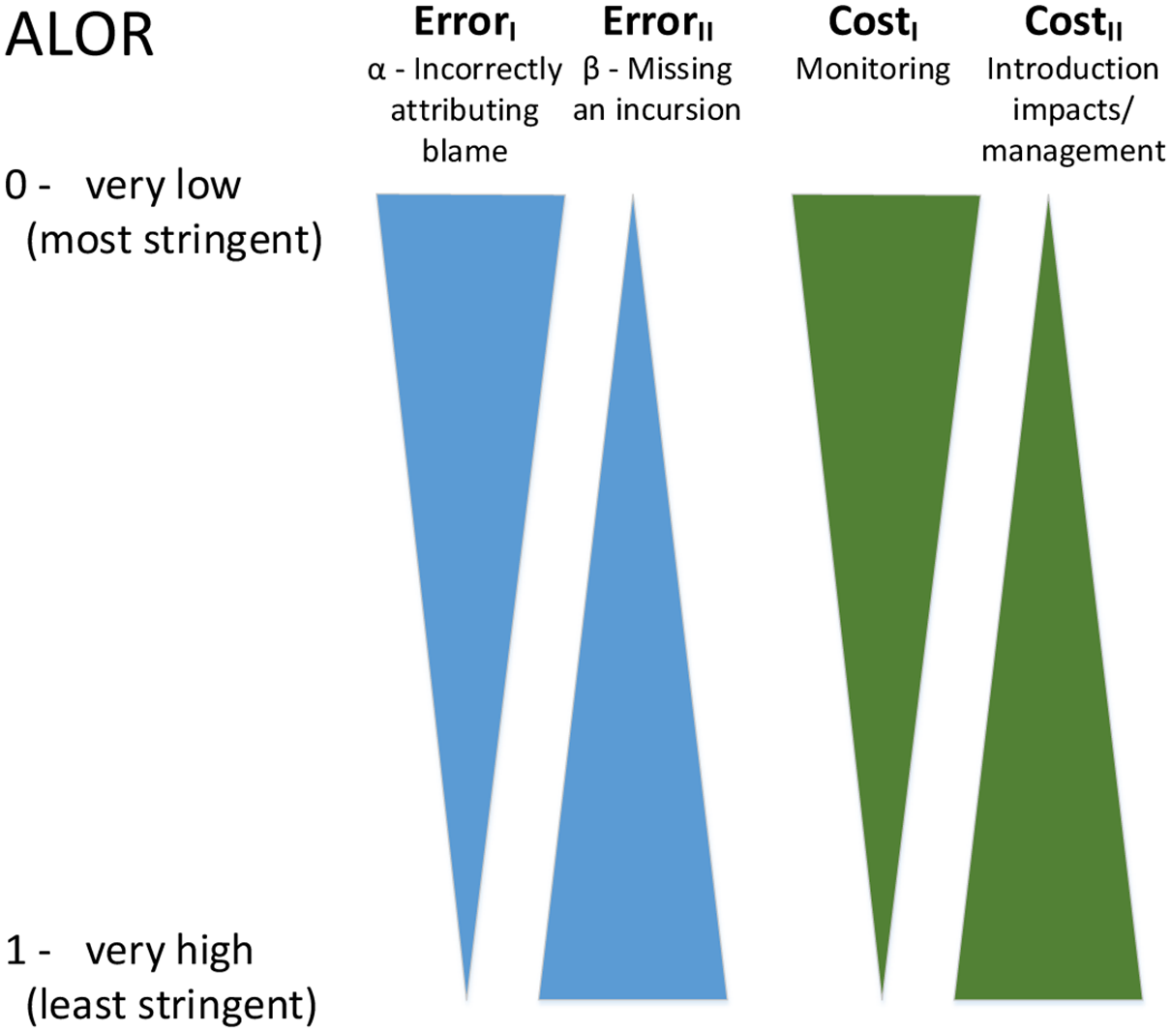
The relationship between ALOR and Type I and Type II errors and costs. The width of the error triangles represents the relative emphasis placed on the two error types, at a given ALOR. The width of the cost triangles represent the actual costs associated with a given ALOR.

We acknowledge that a US$323 million budget is likely unrealistic, at least in the short term. In this case, when an increase in C_I_ sufficient to bring k and k’ to parity is not possible, several options are available: raising the ALOR, lowering the acceptable Type I error rate, or reducing the overall risk. For this case study, raising the ALOR to a lake risk classification of “moderate” (rather than “very low” or higher; e.g., a β of 0.5) would reduce the optimal budget to $6.46 million (Cost_I_ = Cost_II_/k’, US$64,615,600/10, where k’ = Error_II_/Error_I_ = 0.5/0.05). Fig 2 provides an illustration of how modifying the ALOR emphasizes relative error types and effects actual costs. That is, as ALOR decreases the emphasis shifts from avoiding under-management toward potential over-management, and the costs of associated management actions increase.

The second option (lowering the acceptable Type I error rate, e.g, from 0.05 to 0.005) would reduce the optimal budget to $32.3 million (Cost_I_ = Cost_II_/k’, US$64,615,600/2, where k’ = Error_II_/Error_I_ = 0.01/0.005). Similar to the ALOR, setting the acceptable Type I error rate is a decision that will depend on the situation and stakeholders involved. As stated in Methods, we use 0.05 due to the precedence in statistical analysis, but the desired value may vary in a management context. Finnoff et al. [37] looked at the trade-off between prevention or control effort, risk and uncertainty. They found managers tended to avoid prevention efforts because the associated productivity is less certain than the productivity associated with control efforts. This behaviour is analogous to raising ALOR in this study and will result in additional introductions and negative ecological and social impacts.

Given the drawbacks of increasing ALOR (costs of under-management and public perception), explicitly identifying the consequences of insufficient budgets may improve the likelihood of policy makers and stakeholders to ameliorate those shortfalls. It should be noted that many monitoring programs include more than one species. For example, the current MI DEQ monitoring program includes 12 macrophyte species, which with this adjusted budget of US$6.46 million would equate to US$538,000 per species. Other single-species efforts cost up to US$10 million (*Lymantria dispar dispar* Linnaeus, 1758, gypsy moth [38] or US$20 million (*Petromyzon marinus* Linnaeus, 1758, sea lamprey; Nicholas Johnson, USGS, personal communication).

The third option to reconcile the difference between available funds and desirable risk outcomes is to reduce the overall risk by reducing the likelihood of an incursion. For the recreational boating vector, this would require reducing the number of lakes at risk through decisions such as closing associated boat launches to prevent spread by recreational boaters [39,40]. While uncommon, establishing a long-term quarantine to restrict access and prevent the spread of invasive species does occur (e.g., Deep Quarry Lake in Illinois following introduction of *Dreissena polymorpha* Pallas, 1771, zebra mussel, in 2009). A second option is to enhance prevention through active management by installing permanent and mandatory boat wash stations at these lakes [41]. A less-costly prevention option is to encourage and train lake users to perform well-established cleaning behaviours [42].

### Strengths

Decisions on how (best) to effectively leverage available resources are often based on cost-benefit analyses e.g., [6,12]. Studies have shown that prevention provides net economic benefits [43] and even how much prevention is warranted [6]. An additional approach is the use of economic policies to minimize rates of species’ introductions, e.g., using tariffs to internalize the external costs currently imposed by nonindigenous species introductions [44]. These approaches generally place the emphasis on minimizing Type I errors (i.e., management costs) and do not consider policy mandates such as ALOR. For example, in situations with budget constraints, Hauser & McCarthy [12] suggest using the relative probabilities of occurrence and benefits of detection to optimize site selection. However, while consideration of these parameters will improve decision-making, this approach does not consider ALOR and may be missing sites with a probability score that is relevant to policy or management. By making ALOR a driving factor in program design, this framework increases the political and managerial relevance of analyses that also incorporate probability of species presence, expected benefits and surveillance efficacy.

In addition to moving consideration of the errors and costs of under-management to the forefront of program design, this framework has several other important qualities. First, it can be applied to other components of nonindigenous species management—prevention efforts (e.g., ballast discharge standards), control (e.g., number and size of sites), and experimental design (e.g., choice of α, β and effect size in impact assessment studies)–and also adapted for use in other environmental fields such as species conservation or habitat restoration. Along with these other applications is the ability to modify the parameter values. That is, acceptable Type I and II error rates will depend on the situation and associated stakeholder priorities. Second, it provides a transparent process that can strengthen the relationship and potential collaboration between research and management sectors. Defining ALOR will facilitate the translation of management objectives into experimental design, and in turn, improve uptake of those research outcomes for decision-making [45]. Finally, it meets WTO SPS Agreement mandates. Any framework used to develop biosecurity measures must also be in accord with trade mandates, namely those of the WTO SPS Agreement. The WTO SPS Agreement allows Members national sovereignty in setting ALOR and associated trade measures. However, the development of the trade measures must be transparent and based on scientific principles. In explicitly identifying parameters used in risk-related decision making, this framework satisfies those requirements.

### Limitations

The largest limitation in this case study, and likely in other applications, is the uncertainty surrounding the cost analysis. Ideally, a full cost analysis would include not only market costs, but also the many non-market costs (impacts) to environmental, social and cultural values e.g., reduced biodiversity and tourism; see also [46]. However, cost analyses for nonindigenous species have been very limited, calculated only for the direct market costs of a few high-profile invaders and over limited temporal and spatial scale [47]. Often even these are anecdotal in nature, failing to incorporate economic theory [48]. The only cost estimates available and relevant to this study included one indirect market cost (property value decline) and one marginal cost (control); given this, the cost is almost certainly an underestimate. More accurate cost estimates would improve analyses of expected benefits from monitoring or eradication efforts, and facilitate more comprehensive design of surveillance programs. However, incomplete cost estimates are the reality, and can still be used to make management decisions [47].

This study makes several assumptions. First, it assumes the costs of a new introduction would be similar to the cost of currently established species (primarily Eurasian watermilfoil). This is unknown, but given similar mechanisms of impact, this is likely. Second, it assumes the monitoring program protocol leads to 100% detection rates. While this is unlikely, we were unable to find published data on detection rates for inland lake macrophyte monitoring. Thus, applying lower detection rates would be unjustified and lead to a less conservative outcome. In additional to further cost analyses, we suggest this as an area for future research, as it is an important component in optimizing surveillance effort e.g., [12]. A third assumption is that the control and property value costs would be similar for each lake. While this is unlikely, as each lake will have a different size of infestation due to lake size, depth profile and nutrient regime, the control cost (US$102,350) used here was taken over such a range and should approximate an average cost. The property value decline will also depend on perception of the species. While most macrophytes are viewed as unpleasant, several aquatic species may be considered aesthetically valuable (e.g., *Butomus umbellatus* L., flowering rush) and therefore will not decrease property value. A final assumption is that all lakes in this study were either not already invaded by a macrophyte (and hence had not experienced a property value decline) or that an additional introduced macrophyte would lead to an additional 13% property value decline.

In conclusion, the incorporation of ALOR, and by extension this framework, to support decision-making within the realm of biosecurity ensures that management plans account for policy directives, as well as environmental, economic and sociocultural impacts. This case study demonstrates the resources necessary to realize near-zero risk outcomes. Using this framework to transparently and objectively reveal the discrepancies between available resources and acceptable risk will facilitate discussion between policy, management and research sectors, leading to improved biosecurity risk management.

## Supporting Information

S1 Map Map showing location of twelve Michigan lake groups that fund control of aquatic macrophytes through improvement boards, special assessment districts or donations. (PNG)

S1 Supplementary Information 2012 Great Lake Restoration Initiative Action Plan. (PDF)

S2 Supplementary Information The MI DEQ monitoring budget for macrophytes. (DOCX)

S3 Supplementary Information Data related to waterfront properties sold over a three-month period (from March 9-June 9, 2014). (PDF)

S4 Supplementary Information Data related to the cost of species introductions related to control efforts. (DOCX)
