# Supplementary figures and images for: Understanding Acceptable Level of Risk: Incorporating the Economic Cost of Under-Managing Invasive Species
Source: PLoS One. 2015 Nov 4;10(11):e0141958. doi: 10.1371/journal.pone.0141958 (PMC4633185; doi:10.1371/journal.pone.0141958)

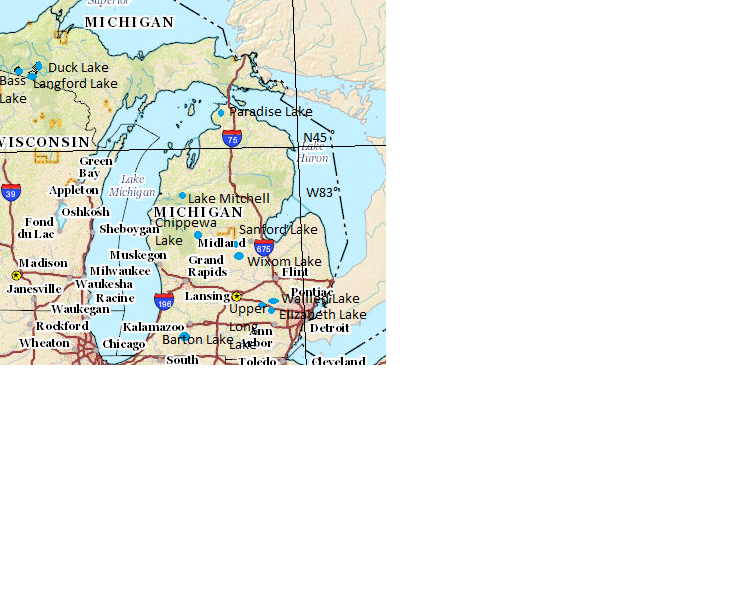

Supplement: S1 Map — (PNG) [file pone.0141958.s001.png]
