# Supplementary material for: Understanding Acceptable Level of Risk: Incorporating the Economic Cost of Under-Managing Invasive Species
Source: PLoS One. 2015 Nov 4;10(11):e0141958. doi: 10.1371/journal.pone.0141958 (PMC4633185; doi:10.1371/journal.pone.0141958)
Supplement: S1 Supplementary Information — (PDF) [file pone.0141958.s002.pdf]

Dated: April 19, 2012
Modified May 9, 2012

U.S. EPA GREAT LAKES NATIONAL PROGRAM OFFICE
Great Lakes Restoration Initiative
2012 Request for Applications

Federal Agency Name: Environmental Protection Agency
Funding Opportunity Title: Great Lakes Restoration Initiative Request for Applications
Announcement Type: Request for Applications
Funding Opportunity Number: EPA-R5-GL2012-1
Catalog of Federal Domestic Assistance (CFDA) Number: 66.469

INTRODUCTION AND OVERVIEW

This Request for Applications (RFA) solicits applications from eligible entities for grants and/or cooperative agreements to be awarded pursuant to the Great Lakes Restoration Initiative Action Plan (http://greatlakesrestoration.us/pdfs/glri_actionplan.pdf). This RFA is EPA's major competitive grant funding opportunity under the Great Lakes Restoration Initiative ("GLRI" or "Initiative") for FY 2012 and is one of several funding opportunities available through federal agencies under the GLRI. Applications are requested for projects identified in the RFA within the following four[1] GLRI focus areas:

- *Toxic Substances and Areas of Concern*[2], including reduction of toxic substances (through pollution prevention or other means) in the most polluted areas in the Great Lakes;
- *Invasive Species*[3], including efforts to institute a "zero tolerance policy" toward new invasions;
- *Nearshore Health and Nonpoint Source Pollution*, including a targeted geographic focus on high priority watersheds and reducing polluted runoff from urban, suburban and agricultural sources; and

---

[1] As described in Section III regarding eligibility, EPA is not requesting and will not consider applications principally pertaining to the remaining GLRI focus area, *i.e.*, Habitat and Wildlife Protection and Restoration. The US Fish and Wildlife Service and the National Oceanic and Atmospheric Administration are handling applications for that focus area this year.

[2] As described in Section III regarding eligibility, EPA is not requesting and will not consider applications principally pertaining to the restoration of Great Lakes Areas of Concern (AOCs). AOC cleanup priorities will primarily be funded through direct contracting, state grants and/or agreements with other federal agencies. Awards pertaining to AOCs will be made at a later date at the discretion of EPA.

[3] As described in Section III regarding eligibility, EPA is not requesting and will not consider applications principally pertaining to Asian carp, which is being addressed pursuant to the FY 2012 Asian Carp Control Framework.

- *Accountability, Education, Monitoring, Evaluation, Communication and Partnerships,* including climate change resiliency and strategic partnerships through Lakewide Management Plans.

Funding/Awards: Approximately $20 million may be awarded as grants and/or cooperative agreements under this RFA for about 100 projects in the four focus areas, contingent upon funding availability, the quality of applications received and other applicable considerations. However, EPA expressly reserves the right to make no awards under a particular focus area or to adjust the number of awards originally anticipated under a specific focus area. Proposed projects should be limited to a project duration of no more than two years. Awards may be fully or incrementally funded. All incrementally funded awards will be subject to the availability of funding, future appropriations, satisfactory performance of work, and other applicable considerations. Applicants may submit more than one application under this RFA provided that each application is for a different project and is submitted separately.

Authorization for GLRI funding is contained in Public Law No: 112-74, under Division E of the EPA provisions identified as *Department of the Interior, Environment, and Related Agencies Appropriations Act, 2012*. EPA has authority to award grants and cooperative agreements for planning, research, monitoring, outreach and implementation projects in furtherance of the GLRI and the Great Lakes Water Quality Agreement. Governmental entities, including state agencies, interstate agencies, Indian tribes, local governments as defined in 40 CFR Section 31.3, institutions of higher learning (*i.e*., colleges and universities), and non-profit organizations are eligible to apply for funding under this RFA. Individuals, foreign organizations and governments, nonprofit organizations exempt from taxation under Section 501(c)(4) of the Internal Revenue Code that engage in lobbying, and "for-profit" organizations are not eligible.

Important Dates:

- Thursday, May 3, 2012 at 2 p.m. Central Daylight Time and Monday, May 14, 2012 at 10:00 a.m. Central Daylight Time - Webinars will be held to discuss the RFA. See Section IV for further information.
- May 24, 2012 –Applications must be submitted to EPA electronically (or through any alternate authorized submission method) through http://www.grants.gov by 11:59 p.m., Eastern Daylight Time. See Section IV for further submission information.
- June, 2012 (tentative) – EPA will begin notifying finalists.
- July, 2012 (tentative) – EPA will begin making official awards.

Other Application Information: For your convenience, an RFA web page has been created at http://www.epa.gov/greatlakes/fund/2012rfa01/ where you will find information relating to the RFA process as well as a link to frequently asked questions (FAQs). We encourage all applicants to sign up for our mailing list and register with us at http://www.epa.gov/greatlakes/maillist . Further submittal information is described in Section IV.

U.S. EPA Great Lakes Restoration Initiative
Request for Applications: EPA-R5-GL2012-1

CONTENTS

I. Application Information ………..…………………………………………………………….4

I.A. Toxic Substances……………………….…………………………………………………...7
I.A.1. Toxic Substance Reduction

I.B. Invasive Species 9
I.B.1. Invasive Species Prevention
I.B.2. Early Detection Technology for High Risk Invasive Species
I.B.3. Invasive Species Control

I.C. Nearshore Health and Nonpoint Source Pollution 13
I.C.1. Making Beaches Safer
I.C.2. Watershed Remediation
I.C.3. Increasing Technical Expertise to Accelerate Nutrient Reduction

I.D. Accountability, Education, Monitoring, Evaluation, Communication, and Partnerships 18
I.D.1. Increasing Climate Change Resiliency in Great Lakes Communities
I.D.2. Implementing Lakewide Management Plan Programs and Projects

II. Award Information 20
III. Eligibility Information 22
IV. Application and Submission Information 25
V. Application Review and Selection Process, including General Criteria and Schedule..................... 36
VI. Award Administration 41
VII. Agency Contacts.. 46
VIII. Other Information 46

Appendix I – Lakewide Management Plan Priorities and Goals I-1
Appendix II – Grants.gov instructions II-1
Appendix III – Budget Sample III-1

U.S. EPA Great Lakes Restoration Initiative
Request for Applications: EPA-R5-GL2012-1

I. APPLICATION INFORMATION

Background, Authority, and Funded Activities:

The President and the U.S. Environmental Protection Agency (EPA), in conjunction with other federal department and agencies, have made restoring the Great Lakes a national priority. The Great Lakes Restoration Initiative ("GLRI" or "Initiative") builds on the prior efforts of federal, state, and local agencies; Indian tribes; businesses; public interest groups; interested citizens; and others to develop a collaborative and comprehensive approach to restoring the Great Lakes. Information about the Initiative can be found at http://greatlakesrestoration.us/. This RFA represents EPA's major competitive grant funding opportunity under the Initiative for FY 2012 and is one of several funding opportunities available through participating federal departments or agencies.

This RFA is expected to result in the award of grants and/or cooperative agreements, as appropriate (hereafter collectively referred to as "grants"), to help implement the GLRI. Authorization for GLRI funding is contained in Public Law No: 112-74, under Division E of the EPA provisions identified as *Department of the Interior, Environment, and Related Agencies Appropriations Act, 2012*.

EPA has authority to award grants for planning, research, monitoring, outreach and implementation projects in furtherance of GLRI and the Great Lakes Water Quality Agreement (GLWQA). The statutory authority to take action to implement the U.S. responsibilities under GLWQA is contained in Section 118(c) of the Clean Water Act. The principal goal of GLWQA is the restoration and maintenance of the chemical, physical, and biological integrity of the Great Lakes ecosystem. Funded activities must advance protection and restoration of the Great Lakes ecosystem in support of (i) the GLRI Action Plan (see http://greatlakesrestoration.us/pdfs/glri_actionplan.pdf), and (ii) EPA's Strategic Plan.[4] For projects with international aspects, the above statutes are supplemented, as appropriate, by the National Environmental Policy Act, Section 102(2) (F).

This RFA solicits applications from eligible entities for grants to be awarded pursuant to the statutory authorities referenced above and the GLRI Action Plan. Up to approximately $20 million may be awarded under this RFA for about 100 projects contingent on the quality of applications received, funding availability and other applicable considerations. Applications are requested for projects identified in the RFA within the following four GLRI focus areas:

---

[4] See EPA's Strategic Plan for Fiscal Years 2011-2015; Goal 2***:*** *Protecting Americas Waters;* Objective 2: - *Protect and Restore Watersheds and Aquatic Ecosystems (Protect the quality of rivers, lakes, streams, and wetlands on a watershed basis, and protect urban, coastal, and ocean waters);* Sub-objective 4: *Improve the Health of the Great Lakes***.** (See http://www.epa.gov/planandbudget/strategicplan.html)

- *Toxic Substances and Areas of Concern*, including reduction of toxic substances (through pollution prevention or other means) in the most polluted areas in the Great Lakes;
- *Invasive Species*, including efforts to institute a “zero tolerance policy” toward new invasions;
- *Nearshore Health and Nonpoint Source Pollution*, including a targeted geographic focus on high priority watersheds and reducing polluted runoff from urban, suburban and agricultural sources; and
- *Accountability, Education, Monitoring, Evaluation, Communication and Partnerships*, including climate change resiliency and strategic partnerships through Lakewide Management Plans.

All projects will be evaluated as described in Section V. For FY 2012, EPA is particularly interested in projects within each RFA funding category that:

- facilitate the creation of new jobs through the use of a civilian conservation corps model for achieving environmental restoration (See Section V.A.6.);
- promote environmental justice by helping to address disproportionate environmental impacts on communities (see Section V.A.4) and/or engage and work with community-based organizations (as defined in this RFA) and other appropriate parties to address the concerns of local communities. (See Section V.A.4.)

Minority Academic Institutions:

Eligible applicants, as defined in Section III, including Minority Academic Institutions (MAIs) as described below, are strongly encouraged to apply for funding under this competition. For purposes of this solicitation, the following are considered MAIs:
1. Historically Black Colleges and Universities, as defined by the Higher Education Act (20 U.S.C. Sec. 1061). A list of these schools can be found at: http://www2.ed.gov/about/inits/list/whhbcu/edlite-list.html;
2. Tribal Colleges and Universities, as defined by the Higher Education Act (20 U.S.C. Sec. 1059(c)). A list of these schools can be found at: http://www2.ed.gov/about/inits/list/whtc/edlite-tclist.html ;
3. Hispanic-Serving Institutions (HSIs), as defined by the Higher Education Act (20 U.S.C. Sec. 1101a(a)(5). There is no list of HSIs. HSIs are institutions of higher education that, at the time of application submittal, have an enrollment of undergraduate full-time equivalent students that is at least 25% Hispanic students at the end of the award year immediately preceding the date of application for this grant; and
4. Asian American and Native American Pacific Islander-Serving Institutions (AANAPISIs), as defined by the Higher Education Act [20 U.S.C. Sec. 1059g(a)(2)]. There is no list of AANAPISIs. AANAPISIs are institutions of higher education that, at the time of application submittal, have an enrollment of undergraduate students that is not less than 10 % students who are Asian American or Native American Pacific Islander.

Multiple Applications:

Applicants may submit more than one application under this RFA provided that each application is for a different project and is separately submitted. Each application must address only one category in one focus area. Except for RFA category I.C.2 (Watershed Remediation), EPA will consider applications that request funding amounts above the EPA-estimated maximum individual project amounts identified in Section I, although EPA does encourage applicants to submit applications that are at or below these estimated maximum project amounts. Categories of eligible activities under each focus area are described in Section I. The anticipated award amounts and the relative allocations for categories of activities are approximations that are subject to change based upon a number of considerations including, but not limited to, EPA's determination that different amounts or allocations would better advance protection and restoration of the Great Lakes ecosystem, funding availability, and other applicable considerations.

Subawardees and/or Contractors:

If you name subawardees/subgrantees and/or contractor(s), including individual consultants, in your application as partners to assist you with the proposed project, pay careful attention to the information in Section III regarding "Coalitions" and Section IV. I (Questions and Answers Pertaining to Contracts and Subawards) .

RFA Terms:

For purposes of this RFA:

1. The term "**climate change**" means the impacts of climate change on the Great Lakes. This RFA is intended to call attention to the impacts of climate change on the Great Lakes as well as adaptation efforts needed to respond to those impacts. The long term results of many GLRI projects may be affected by the degree to which applicants incorporate an understanding of how the impacts of climate change may influence their work. Applicants are encouraged to consider their projects' vulnerabilities to climate change impacts and how to address those vulnerabilities. For more information on the human health and environmental effects of climate change, visit: http://www.epa.gov/climatechange/. For more information on climate change adaptation, visit: http://www.epa.gov/climatechange/effects/adaptation.html.

2. The term "**community-based organization**" means a non-governmental organization that has demonstrated effectiveness as a representative of a community or a significant segment of a community and that helps members of that community or segment obtain environmental, educational, or other social services. A community-based organization must be a nonprofit or not for profit corporation in good standing under state or tribal law with authority to enter into binding legal agreements. The community-based organization need not be tax-exempt under the Internal Revenue Code but may use documentation of tax-exempt status to demonstrate that it is a nonprofit. Nonprofit organizations exempt from taxation under section 501(c)(4) of the

Internal Revenue Code that engage in lobbying are not eligible to receive grants or subgrants under this RFA. (See Section V.A.4.)

3. The term "**output**" means an environmental activity, effort, and/or associated work product related to an environmental goal and objective that will be produced or provided over a period of time or by a specified date. Outputs may be quantitative or qualitative but must be measurable over the term of a grant funding period.

4. The term "**outcome**" means the result, effect or consequence that will occur from carrying out an environmental program or activity that is related to an environmental or programmatic goal or objective. Outcomes may be environmental, behavioral, health-related, or programmatic in nature, must be quantitative, and may not necessarily be achievable within a grant funding period.

5. The term "**Indian Tribe**" includes: Indian/Native American Tribal Government (Federally Recognized), Indian/Native American Tribal Government (Other than Federally Recognized) and Indian/Native American Tribally Designated Organization.

Categories of Funding Opportunities:

Applications are requested for the categories of activities described below for the respective focus areas. Applications must address only one category within a focus area.

Category I.A. - Request for Applications - Toxic Substances.

General Background: While levels of certain persistent toxic substances (*e.g.*, mercury) have been significantly reduced in the Great Lakes ecosystem over the past 30 years, these chemicals continue to be present at levels above those considered safe for humans and wildlife, warranting fish consumption advisories in all five Great Lakes and the connecting channels. Indigenous communities and others who frequently consume Great Lakes fish are most at risk from fish contamination. Continuing sources of persistent toxic substances include releases from contaminated sediments; industrial and municipal point sources; nonpoint sources including atmospheric deposition, agricultural and urban runoff; contaminated groundwater; and cycling of these substances within the Lakes.

In addition to the well-known persistent toxics like polychlorinated biphenyls (PCBs), mercury, and banned pesticides, chemicals of emerging concern have been detected in the Great Lakes over the past several years that may pose threats to the health of the ecosystem. Some such chemicals are found in very common products, *e.g.*, pharmaceuticals and personal care products, yet there is very little environmental information about the effects of these chemicals. To protect human and ecosystem health against future threats, these substances must be better understood with respect to their potential hazards, routes of exposure, and any effective preventative or remedial actions.

Category I.A. - Toxic Substance Reduction: EPA expects to provide approximately $1,000,000 for approximately 10 to 15 projects in amounts up to $200,000 for Great Lakes toxic substance reduction activities, including pollution prevention and human health projects. Projects should aim to reduce the amount of toxic substances in or entering into the Great Lakes ecosystem and/or reduce the risk of exposure to toxic substances from Great Lakes fish consumption (especially with respect to sensitive populations such as women of child-bearing age, children under 15, and populations that rely upon fish or other native foods for subsistence). Projects submitted under this category must include one or more of the following:

- Actions delivering reductions of toxic substances targeted by the Great Lakes binational Toxics Strategy and/or the EPA Chemical Action Plans (http://www.epa.gov/opptintr/existingchemicals/pubs/ecactionpln.html). Applicants should include a link to or name the applicable contaminants.
- Actions supporting reductions in the use of mercury in products, better management of mercury-containing wastes, or reductions in mercury emissions. During the evaluation process described in Section V for projects addressing this area, the degree to which the project helps states, Indian tribes, and local governments implement the recommendations of the Great Lakes Regional Collaboration's *Mercury in Product Phase-down Strategy* (http://glrc.us/documents/MercuryPhaseDownStrategy06-19-2008.pdf) and/or the Great Lakes Regional Collaboration's *Mercury Emissions Strategy* (http://glrc.us/documents/MercuryEmissionsPhaseDownStrategy12-07-2010.pdf) may be more favorably considered under the evaluation criteria for "Project Summary and Approach". (See Section V.A.1.)
- Actions that will reduce the exposure of toxic substances, such as mercury, to the fish-consuming Great Lakes population, especially sensitive populations, so that levels of these toxic substances in the human body are minimized.
- Actions to improve the public's understanding of the risks and benefits of consuming fish. These projects are expected to improve and enhance current state fish advisory programs and could include monitoring, evaluation of risks and benefits of consuming fish, and communication to the public.

Ineligible activities under this category include:

- remedial actions to address contaminated sediment, soil, debris, or other contaminated materials;
- erosion control measures; and
- human health projects that are not managed directly by state or tribal fish consumption advisory programs or by a partner of the state or tribal fish consumption advisory program. A letter of support from the applicable state or tribal health advisory program demonstrating support for the project must be included with the submission and attached as described in Appendix II, item 11.

See Section III for other ineligible activities.

Outputs from the projects under this category should include one or more of the following and must link to the GLRI Action Plan's Measures of Progress or goals and objectives:

- The removal of measurable amounts (pounds) of toxic substances from the Great Lakes ecosystem.
- The prevention of measurable amounts (pounds) of toxic substances from being released into the Great Lakes ecosystem.
- The protection of Great Lakes fish consumers from harmful chemicals such as mercury and PCBs, with sound and sensible advice provided through enhanced and expanded state and tribal fish advisory programs.
- An increased effort of the Great Lakes medical and health communities to educate the general public regarding the benefits and risks of Great Lakes fish consumption.
- An increase and improvement in spatial coverage of monitoring of legacy chemicals in fish in the Great Lakes basin.
- The development of new chemical protocols for evaluating fish consumption risks.

Applicants must also demonstrate how their proposed project will achieve one or more of the following outcomes:

- The entry of fewer toxic substances into the Great Lakes ecosystem.
- The increased safe reuse and recycling of toxic materials.
- The virtual elimination of the release of any or all persistent toxic substances to the Great Lakes ecosystem.
- The significant reduction of exposure to toxic substances from historically-contaminated sources through source reduction and other exposure reduction methods.
- The reduction of levels of toxic chemicals to the point that restrictions on the consumption of Great Lakes fish can be lifted.
- The protection of the health of Great Lakes basin residents from adverse effects associated with the presence of toxic substances in the Great Lakes ecosystem.
- The protection of the health and integrity of wildlife populations and habitat from adverse chemical and biological effects associated with the presence of toxic substances in the Great Lakes ecosystem.
- The prevention and/or reduction of toxic substances from accumulating in the bodies of Great Lakes residents, with a focus on sensitive populations.

Category I.B. - Request for Applications - Invasive Species.

General Background: Progress toward restoring the Great Lakes has been significantly undermined by the effects of non-native invasive species. Over 180 non-native species now exist in the Great Lakes. The most invasive of these propagate and spread, ultimately degrading

habitat, out-competing native species, and short-circuiting food webs. New invasive species can be introduced into the Great Lakes region through various pathways, including: commercial shipping, canals and waterways, trade of live organisms, and activities of recreational and resource users. Efforts must be accelerated to prevent new introductions of invasive species and to minimize the further spread of the organisms to inland lakes, the Mississippi River watershed, and beyond.

Prevention is the most cost-effective approach to dealing with organisms that have not arrived but which could potentially threaten the Lakes. If new invasive species are detected when their populations are small, rapid response can help prevent these populations from becoming established. In addition to conventional netting and trapping, molecular/genetic techniques such as eDNA (detection and quantification of environmental DNA obtained directly from small water samples of lakes, ponds and streams) can help predict the occurrence of species in the aquatic environment. Once invasive species become established in the Great Lakes, they are virtually impossible to eradicate. However, in some cases, they can be controlled to reduce impacts on the Great Lakes ecosystem.

Federal agencies need to work with state, tribal, and local governments, academic institutions, industry, and non-governmental organizations to:

1. Stop the introduction of new invasive species into the Great Lakes through enhanced prevention programs;
2. Enhance the ability to detect invasive species at low population levels to facilitate rapid response actions; and
3. Control and reduce the spread of invasive species already in the Great Lakes ecosystem, through enhanced on-the-ground and in-the-water efforts.

To address these issues and make progress towards achieving the goals, objectives, and targets identified for the Invasive Species focus area in the GLRI Action Plan, EPA is soliciting applications for projects as described below.

Category I.B.1. - Invasive Species Prevention: EPA expects to provide approximately $3.3 million for approximately 8 to 15 projects in amounts up to $400,000 that will prevent new introductions of invasive species into the Great Lakes ecosystem. Projects should prevent introductions of live invasive species through domestic commerce (including internet sales of plants and animals) and international trade (including the accidental importation of unintended species); reduce the spread of invasive species via canals and waterways; and/or promote safe recreation and resource use. Prevention efforts may use well-established techniques and/or demonstrate the use of innovative technologies to achieve results.

Applications should describe a strategic approach that: identifies relevant vectors of introduction; estimates the significance of the vector to the project area; estimates the net impact of the proposed prevention activities on the vector; uses a multi-organizational approach to implementation, including working with relevant governmental agencies, where appropriate;

demonstrates measurable progress; includes public stewardship opportunities; and helps establish prevention efforts that will continue beyond the project period. Applicants may develop prevention approaches using information from risk assessments, including those funded by the GLRI (See, *e.g.*, the *GLANSIS Watch list of Potential Great Lakes Aquatic Invasive Species* at http://www.glerl.noaa.gov/res/Programs/glansis/watchlist.html, and the *GLMRIS* analysis of species currently within the Mississippi River watershed but not in the Great Lakes, at http://glmris.anl.gov/documents/ans/index.cfm).

Outputs from the projects under this category should include the following and must link to the GLRI Action Plan's Measures of Progress or goals and objectives:

- Completion of projects that implement prevention measures for vectors/pathways including, but not limited to (1) preventing the release of live invasive species in domestic commerce and international trade; (2) reducing the spread of invasive species through canals/waterways; and/or (3) promoting safe recreation and resource use.

Applicants must also demonstrate how their proposed project will achieve one or more of the following outcomes:

- The reduction in the rate of non-native species being newly detected in the Great Lakes ecosystem.
- The prevention of the spread of invasive species beyond their current range by means of recreational activities and canals and waterways.
- The evaluation and minimization of the risk of introduction of species which are imported for various uses into the Great Lakes ecosystem.

Category I.B.2. - Early Detection Technology for High Risk Invasive Species: EPA expects to provide approximately $1.8 million for approximately 3 to 5 projects in amounts up to $600,000 to advance development of technology based on molecular/genetic techniques that is capable of detecting invasive species at low population numbers. Risk assessments are helping prioritize early detection efforts and have led to the development of watch lists for high-risk organisms to the Great Lakes. (See, *e.g.*, the *GLANSIS Watch list of Potential Great Lakes Aquatic Invasive Species* at http://www.glerl.noaa.gov/res/Programs/glansis/watchlist.html, and the *GLMRIS* analysis of species currently within the Mississippi River watershed but not in the Great Lakes, which can found at http://glmris.anl.gov/documents/ans/index.cfm). New technologies that are especially needed are those that can detect high-risk species such as northern snakehead (*Channa argus*), hydrilla (*Hydrilla verticillata*), golden mussel (*Limnoperna fortunei*), various Ponto-Caspian water fleas including *Daphnia cristata*, and killer shrimp (*Dikerogammarus villosus*).

Applications should describe a strategic approach to develop or refine technology that: includes an overview of the molecular/genetic technique that will be used; identifies the species to be targeted; summarizes the risk posed by the targeted species; describes the practical application of the technology to detect species in the Great Lakes basin; provides a clear timeline for the

development process; anticipates possible development difficulties; describes how effectiveness will be assessed; demonstrates use within mesocosms or within the Great Lakes basin; and works with relevant private entities and governmental agencies, where appropriate.

Outputs from the projects under this category should include one or more of the following and must link to the GLRI Action Plan's Measures of Progress or goals and objectives:

- Development or refinement of molecular/genetic technology that can be used to detect high-risk species within the Great Lakes basin.
- Assessment of the longevity of the applicable genetic/molecular marker in the environment and appropriate interpretations for positive detections.
- Assessment of the sensitivity of the technology and/or relationship between detection signal strength and actual population size.
- Applied use of the technology within mesocosms or within the Great Lakes basin.

Applicants must also demonstrate how their proposed project will achieve one or both of the following outcomes:

- The improved ability to detect invasive species in the Great Lakes and the compilation and provision of up-to-date critical information needed by decision makers for evaluating potential rapid response actions.
- The enhanced surveillance of coastal ecosystems along the U.S. Great Lakes shore for invasive species.

Category I.B.3. - Invasive Species Control: EPA expects to provide approximately $2.3 million for approximately 5 to 10 projects in amounts up to $400,000 to eradicate and control invasive species already present in the Great Lakes ecosystem. Projects should implement on-the-ground and/or in-the-water invasive species control actions.

Applications should describe a strategic approach that: identifies the ecological significance of the project location; estimates the short and long-term ecological benefits of the proposed effort; uses a multi-organizational approach to implementation, including working with relevant private entities and governmental agencies, where appropriate; demonstrates measurable progress; includes public stewardship opportunities; and establishes efforts that will help maintain the project site beyond the project period. Control efforts may use well-established techniques and/or demonstrate the use of new or innovative technologies to achieve results.

Outputs from the projects under this category should include one or more of the following and must link to the GLRI Action Plan's Measures of Progress or goals and objectives:

- Control of invasive species populations in the Great Lakes ecosystem, as measured by acres managed or quantity of invasive species removed (total number of individuals or total pounds).
- Implementation of multi-agency rapid response plans.

- Implementation of projects that demonstrate innovative control measures.

Applicants must also demonstrate how their proposed project will achieve one or more of the following outcomes:

- An enhanced capability by jurisdictional authorities to effectively respond to new detections of invasive species.
- The rapid control, reduction, and/or eradication of invasive species populations within the Great Lakes ecosystem using site-specific rapid response plans.
- The rapid control, reduction, and/or eradication of invasive species populations within the Great Lakes ecosystem consistent with existing fish, wildlife, and other ecosystem component management plans/strategies.

Category I.C. - Request for Applications - Nearshore Health and Nonpoint Source Pollution.

General Background: The nearshore environment includes both aquatic and terrestrial features that extend variable distances away from the land-water intersection. This is the area in which most residents and visitors interact with the Great Lakes. The nearshore waters of the Great Lakes are a primary source of drinking water, supplier of fish for personal and commercial benefit, and a recreational outlet for millions of U.S. residents and visitors. Nearshore water quality has become degraded, as evidenced by eutrophication resulting from excessive nutrients; harmful algal blooms; mats of *Cladophora* on beaches; avian botulism; and beach closings. Causes of these problems include excessive nutrient loadings from both point and nonpoint sources, bacteria and other pathogens, development and shoreline hardening, agricultural practices, failing septic systems, improper grey-water discharge, inadequate pump-out stations for recreational boats, and invasive species.

Nonpoint sources are now the primary contributors of many pollutants to the Great Lakes and their tributaries. Although some nonpoint sources act on a whole-basin scale (*e.g.*, atmospheric deposition of toxic substances) many smaller-scale sources contribute to degraded water quality in Great Lakes tributaries and nearshore waters. The complexity of the pollutants and their presence in soil, water and air make pollution abatement for nonpoint sources particularly difficult to address. Control strategies to date have been inadequate to deliver the degree of stream and lake restoration necessary for the protection and maintenance of the Great Lakes. Implementation of best management practices can have multiple benefits, including simultaneous reductions in runoff of soils, nutrients, pesticides, and other nonpoint source pollutants.

The GLRI Action Plan identifies five targeted geographic watersheds (Fox River, Saginaw River, Maumee River, St. Louis River, and Genesee River) for nonpoint source pollution control measures. Three of these targeted watersheds (Maumee River, Saginaw River, and Lower Fox River) have been clearly identified as watersheds with excessive phosphorus inputs, the

occurrence of harmful algal blooms, or the occurrence of nuisance algae (*Cladophora*) in the corresponding nearshore areas. Starting in FY 2012, the GLRI Inter-agency Task Force has identified smaller priority sub-watersheds located within these larger targeted watersheds for coordinated phosphorus reduction efforts based on the existence of watershed management plans, percentage of agricultural land, potential for high impact phosphorus reduction practices, and local interest. The priority sub-watersheds are:

Maumee River – Upper Blanchard River Watershed
Lower Fox River– Upper East River and Upper Duck Creek
Saginaw River – Swartz and Kearsley Creek Watersheds

To address these issues and make progress towards achieving the goals, objectives, and targets identified for this focus area in the GLRI Action Plan, EPA is soliciting applications for projects as described below.

Category I.C.1. - Making Beaches Safer: EPA expects to provide approximately $3.9 million for approximately 7 to 35 projects in amounts up to $500,000 to implement actions to reduce or eliminate sources of bacteriological, algal or chemical contamination that have been identified through, or are consistent with, sanitary surveys at Great Lakes beaches.

EPA is requesting applications for projects that will reduce or eliminate the sources of bacteriological, algal or chemical contamination identified at Great Lakes beaches. EPA especially encourages projects to be performed in the U.S./Binational Areas of Concern (listed at http://www.epa.gov/glnpo/aoc/) such that any project will assist in removal of Beach Closing (including recreational contact) Beneficial Use Impairments (BUIs).

Projects should be designed to reduce risk to human health at Great Lakes swimming beaches by developing and implementing projects to reduce, manage, or eliminate contamination sources that have been identified through the use of beach sanitary surveys, such as those funded in EPA's FY10 GLRI Request for Proposals, or equivalent documentation. Projects should include a description of how the long-term sustainability of such projects for one location can be assured or how projects can have value at more than one beach location. Applicants should specify the problem to be resolved as well as the proposed solution as requested in Section IV.C.2.b.i. Projects submitted under this category may include one or more of the following:

- Installation of green stormwater infiltration systems near Great Lakes beach areas with persistent problems.
- Implementation of green stormwater infrastructure approaches that can reduce, capture, and treat stormwater runoff at its source.
- Reduction or removal of impervious surfaces near beach areas.
- Re-grading of beach areas and/or adjacent parking lots.
- Diversion/filtration of stormwater runoff at beach areas through installation of swales or rain gardens.

- Implementation of permanent waterfowl management measures to prevent landing/congregation at beaches.

Outputs from the projects under this category should include one or more of the following and must link to the GLRI Action Plan's Measures of Progress or goals and objectives:

- Reduction in the number of pollution sources impacting Great Lakes beaches.
- Reduction in the number of Great Lakes beach closures or advisories issued.
- Documentation of mitigation measures taken and outcomes achieved which can be applied at other Great Lakes beaches.
- Reduction in nuisance algal blooms and ambient water concentrations of nitrogen and phosphorus in Great Lakes coastal areas.

Applicants must also demonstrate how their proposed project will achieve one or more of the following outcomes:

- Water quality is improved at Great Lakes beaches due to reductions in bacteriological, algal, and chemical contamination.
- Protection of public health is improved at Great Lakes beaches.
- Holistic watershed approaches to Great Lakes beach management are implemented, resulting in a more efficiently directed beach program.

Category I.C.2. - Watershed Remediation: EPA expects to provide approximately $6 million for approximately 6 to 25 projects to accelerate watershed remediation. The maximum amount of federal funds that will be awarded for any project in this category is $1 million and applications cannot ask for more than that. As noted in Section V, EPA may view more favorably proposals for watershed remediation projects located in the geographic areas targeted in the GLRI Action Plan (Fox River, Saginaw River, Maumee River, St. Louis River, and Genesee River) for nutrient nonpoint source pollution control measures. In addition, watershed remediation projects located in the priority sub-watersheds of the Maumee River (Upper Blanchard River Watershed), the Lower Fox River (Upper East River and Upper Duck Creek), and the Saginaw River (Swartz and Kearsley Creek Watersheds) may be viewed more favorably than others.

To be eligible under this category, applications must demonstrate how the project will:

- Accelerate watershed remediation in one or more watersheds of the Great Lakes basin;
- Implement best management practices and management measures contained in state-approved *Nine-Element* Watershed Management Plans, TMDL implementation plans, or in other watershed management plans that are consistent with the components outlined in Section 2.6 of EPA's *Handbook for Developing Watershed Plans to Restore and Protect Our Waters* (http://water.epa.gov/polwaste/nps/handbook_index.cfm);

- Identify specific components of such a plan that would be implemented within three years; and
- Estimate the expected environmental result for each best management practice or management measure from the three-year implementation period.

Watershed-based plans that are consistent with the components outlined in EPA's *Nine Elements Guidance* are designed to address documented nonpoint source-related water quality problems and to help prevent future nonpoint source water quality-related problems. Such plans are based upon sound science and evaluation techniques; have measurable outcomes; are developed with stakeholder/public involvement; and leverage additional resources. The approved *Nine-Element* Watershed Management Plans can be found at:

- Illinois: http://www.epa.state.il.us/water/watershed/reports/biannual-319/2011/september.pdf
- Indiana: http://www.in.gov/idem/nps/3180.htm
- Michigan: http://www.michigan.gov/deq/0,4561,7-135-3313_3682_3714-95955--,00.html
- Minnesota: http://www.pca.state.mn.us/index.php/water/water-types-and-programs/surface-water/watershed-approach/watershed-management-plans.html
- New York: http://www.dec.ny.gov/chemical/23844.html
- Ohio: http://www.dnr.state.oh.us/H_Nav2/Water/WatershedCoordinator/tabid/9192/Default.aspx
- Pennsylvania: http://www.portal.state.pa.us/portal/server.pt?open=514&objID=554271&mode=2
- Wisconsin: http://dnr.wi.gov/water/watersheds/

Applicants should provide a map that delineates the project boundaries within the selected watershed and identifies the critical areas where work will be implemented. Applicants should also plan to estimate the impact of ongoing implementation through a Spreadsheet Tool for Estimating Pollutant Load (STEPL), which employs algorithms to calculate the load reductions that would result from implementation of various best management practices.

Ineligible activities under this category include monitoring activities. GLRI-funded watershed projects will instead be incorporated into the applicable state's ongoing nonpoint source monitoring efforts.

Outputs from the projects under this category should include one or more of the following and must link to the GLRI Action Plan's Measures of Progress or goals and objectives:

- Reduction in nutrient inputs (source reduction and/or loadings) to Great Lakes tributaries and nearshore waters.
- Reduction in sediment inputs (source reduction and/or loadings) to Great Lakes tributaries and nearshore waters.
- Reduction in concentrations of soluble reactive phosphorus in major Great Lakes tributaries, especially during critical spring and summer storm events.

- Removal of BUIs and/or delisting of Clean Water Act Section 303(d) impairments in the Great Lakes.
- Reduction in the number of incidents of harmful algal blooms, avian botulism, and/or excessive *Cladophora* growth (as compared to the number of incidents in 2008 for all Lakes except Lake Michigan, for which 2007 shall be used) in the Great Lakes ecosystem.
- Increase in the percentage of agricultural lands in the Great Lakes ecosystem comprehensively managed to reduce nutrient export utilizing a CORE 4 approach promoting soil health.

Applicants must also demonstrate how their proposed project will achieve one or more of the following outcomes:

- Soil erosion and the loading of sediments, nutrients, and pollutants into Great Lakes tributaries are significantly reduced.
- Nearshore Great Lakes aquatic resources are protected/enhanced/restored.
- Trophic status and/or biotic integrity in Great Lakes stream or lake water quality are improved.

Category I.C.3. - Increasing Technical Expertise to Accelerate Nutrient Reduction: EPA expects to provide approximately $500,000 for approximately 1 to 5 projects in amounts up to $500,000 to increase and ensure long-term adoption of nutrient reduction strategies in agriculturally-dominated watersheds through supporting organized networks of technical experts. To be eligible under this category, applications must complement targeted federal and state efforts to reduce and track soluble reactive phosphorus in the Maumee River, Fox River, and/or Saginaw River basins. Watershed remediation projects located in the priority sub-watersheds of the Maumee River (*i.e.*, Upper Blanchard River Watershed), the Lower Fox River (*i.e.*, Upper East River and Upper Duck Creek), and the Saginaw River (*i.e.*, Swartz and Kearsley Creek Watersheds) may be viewed more favorably than others.

Projects should accelerate ongoing efforts to reduce nutrient losses through direct technical assistance to agricultural producers resulting in the adoption and implementation of nutrient management strategies and practices on the land. Projects should seek to accelerate adoption of nutrient reduction strategies by determining both the environmental and economic benefits of adopting nutrient reduction strategies through direct measurement of these benefits, followed by the quick reporting of these benefits to the wider agricultural community (via, *e.g.*, demonstration farms, year-to-year consultation, workshops, local meetings).

Applicants should also demonstrate a history of working in agricultural settings and/or working one-on-one with farmers on those technical aspects of farm operations that will contribute to water quality protection.

Outputs from the projects under this category should include one or more of the following and must link to the GLRI Action Plan's Measures of Progress or goals and objectives:

- An increase in assistance, information exchange, and consultation among federal/state agencies, non-governmental agencies, academia, technical experts, and farmers.
- An increase in acres and duration of acres adopting nutrient reduction strategies.
- An increase in innovative and advanced nutrient reduction strategies on farmland.
- A compilation and communication of environmental and economic benefits which can accrue to farmers due to adoption of nutrient reduction strategies.

Applicants must also demonstrate how their proposed project will achieve the following outcome:

- A reduction in soluble reactive phosphorus loadings to the Maumee, Fox, or Saginaw rivers and tributaries.

Category I.D. - Request for Applications – Accountability, Education, Monitoring, Evaluation, Communication, and Partnership.

General Background: Effective governmental partnerships, informed public participation, and ecosystem monitoring are important components of a long-term Great Lakes protection and restoration strategy. To address these components and make progress towards achieving the goals, objectives, and targets identified for this focus area in the GLRI Action Plan, EPA is soliciting applications for projects as described below.

Category I.D.1. - Increasing Climate Change Resiliency in Great Lakes Communities: EPA expects to provide approximately $200,000 for 2 to 5 projects ranging in amounts up to $100,000 for Great Lakes communities. This offering is intended to build upon the work completed under the GLRI in 2010 and 2011 by NOAA, USGS and other federal agencies. Their work has been providing climate change information, tools, and training to build the capacity of resource managers and local decision-makers in communities throughout the Great Lakes to understand and address local vulnerabilities to climate change impacts. EPA seeks to fund work in those communities who now have the necessary tools and partnerships in place and are ready to take the next step to address current and projected impacts of climate change on their communities' ecological resources or environmental infrastructure. Applications should focus on development and implementation of vulnerability assessments and adaptation plans to increase the resiliency of the respective community to climate change. This work should help communities ensure that ecological resources (*e.g.*, areas identified as critical habitat) are resilient to climate change, and that environmental infrastructure (*e.g.*, stormwater or wastewater treatment) remains effective as the climate changes.

Projects under this category that seek to accomplish one or more of the following may be evaluated under Section V.A.1 more favorably than others.

- The identification of local vulnerabilities to climate change impacts on specific ecological resources and/or environmental infrastructure in the Great Lakes basin;

- A demonstration of the risks posed by those vulnerabilities which affect the achievement of the goals and objectives of the GLRI;
- The planning, development, and prioritization of actions to reduce those vulnerabilities; and
- The implementation of priority actions that reduce those vulnerabilities and increase the resiliency of ecological resources or environmental infrastructure.

Outputs from the projects under this category should include one or more of the following and must link to the GLRI Action Plan's Measures of Progress or goals and objectives:

- Identification of the specific vulnerabilities faced by the local community and the articulation of feasible options to reduce these vulnerabilities.
- The development of adaptation strategies or plans developed by or in partnership with local Great Lakes resource managers who will need to deal with climate change vulnerabilities (*e.g*., public land owners, utilities, municipalities, *etc*.).
- The implementation of actions to address climate change impacts on community ecological resources and environmental infrastructure, including actions that lead to a reduction in local vulnerabilities.
- The implementation of actions that protect the results of GLRI efforts to improve water quality and the Great Lakes ecosystem from the impacts of climate change.

Applicants must also demonstrate how their proposed project will achieve one or more of the following outcomes:

- Increased capacity of Great Lakes communities to incorporate information on climate change impacts into their planning and decision-making.
- Increased integration of adaptation planning into resource or infrastructure management.
- Increased probability of long-term success of GLRI habitat and watershed projects.
- Decrease in overall vulnerability of Great Lakes communities to climate change impacts.
- Reduction in climate-related risks of increased nonpoint source loadings from tributaries.
- Increase in ecosystem resilience, thereby improving the capabilities of ecosystems to adapt to changes in climate.

Category I.D.2. - Implementing Lakewide Management Plan Programs and Projects: EPA expects to provide approximately $1 million for approximately 3 to 10 projects in amounts up to $300,000 to strategically implement critical Lakewide Management Plan (LaMP) projects not covered by other sections of this RFA that support LaMP priorities described in Appendix I. Proposals should focus on implementation of LaMP priorities. For the purpose of this RFA, the term "LaMP" includes Lakewide Management Plans for Lakes Ontario, Michigan, Erie, and Superior, the Lake Huron Binational Partnership Action Plan, and the Comprehensive Management Plan for Lake St. Clair. (See Appendix I for information about LaMP goals and priorities and other projects.)

Outputs from the projects under this category should include one or more of the following and must link to the GLRI Action Plan's Measures of Progress or goals and objectives:

- Increase in number and/or diversity of stakeholders participating in the development and implementation of LaMP priorities.
- Increase in the implementation of priority LaMP projects.

Applicants must also demonstrate how their proposed project will achieve one or more of the following outcomes:
- Provision of high-quality information on Great Lakes issues to governmental organizations by a more diverse group of Great Lakes stakeholders and citizens.
- An increase in citizen actions that will better protect and restore the Great Lakes ecosystem.
- Implementation of projects that improve water quality and the Great Lakes ecosystem.

II. AWARD INFORMATION

Amounts, Targets, and Number of Projects: Approximately **$20** million is expected to be awarded under this RFA for approximately 100 projects.[5] The number of projects EPA will fund as a result of this RFA will be based on the quality of applications received, the availability of funding, and other applicable considerations. The anticipated total amount of awards to be provided and any anticipated individual project maximum amounts identified in Section I are **estimates** only**,** and are being provided solely for application preparation purposes. (Please note, however, that the maximum amount of federal funds that will be awarded for a Watershed Remediation project per Section I.C.2 is $1,000,000.) While applications requesting funding above any anticipated maximum individual project amounts identified in Section I for the different categories will be considered (except as noted above for applications under I.C.2), EPA encourages applicants to submit applications that do not exceed the anticipated maximum individual project amounts.

The actual award amounts and number of projects awarded under each of the categories and focus areas in Section I may differ from what is estimated for many reasons, including the number of meritorious applications received and funding availability. In addition, EPA reserves the right to increase or decrease (including decreasing to zero) the total number and amount of awards under each focus area (and categories under a focus area), or change the ratio of assistance agreements it awards among the focus areas (and categories under the focus areas).

EPA reserves the right to reject all applications and make no awards under any or all of the categories identified in this RFA or make fewer awards than anticipated.

Anticipated Project Start and End Dates: EPA anticipates that applicants will submit certifications and other documentation required for a full and complete funding package so that their projects could, if selected, proceed expeditiously. Proposed projects should be limited to a

[5] If an applicant submits multiple proposals that are selected for award, one award may cover more than one proposed project if appropriate.

project duration of no more than two years, except for projects submitted for Watershed Remediation (Section I.C.2) which may have a duration of up to three years.

Additional Awards: EPA reserves the right to make additional awards under this RFA, consistent with Agency policy and guidance, if additional funding becomes available after (or at the time) original selections are made. Any additional selections for awards will be made no later than six months after the original selection decisions.

Funding Type: Successful applicants will be issued either a grant or cooperative agreement, as appropriate. A cooperative agreement is an assistance agreement that is used when there is substantial federal involvement with the recipient during the performance of an activity or project. EPA awards cooperative agreements for those projects in which it expects to have substantial interaction with the recipient throughout the performance of the project. EPA will negotiate the precise terms and conditions of "substantial involvement" as part of the award process. Federal involvement may include close monitoring of the recipient's performance; collaboration during the performance of the scope of work; review of proposed procurements in accordance with 40 CFR Sections 30.44(e) and 31.36(g); reviewing qualifications of key personnel; and/or review and comment on the content of printed or electronic publications prepared. EPA does not have the authority to select employees or contractors employed by the recipient. The final decision on the content of reports rests with the recipient.

Future Funding: Selection or award of funding under this RFA is not a guarantee of future funding.

Partial Funding: In appropriate circumstances, EPA reserves the right to partially fund applications by funding discrete portions or phases of proposed projects. If EPA decides to partially fund an application, it will do so in a manner that does not prejudice any applicants or affect the basis upon which the application was evaluated and selected for award, and, therefore, maintains the integrity of the competition and selection process.

III. ELIGIBILITY INFORMATION

Applicant Eligibility (CFDA 66.469): Governmental entities, including state agencies, interstate agencies, Indian tribes, local governments as defined in 40 CFR Section 31.3, institutions of higher learning (*e.g*., colleges and universities subject to 40 CFR Part 30 or 40 CFR Part 31), and nonprofit organizations are eligible to apply for funding under this RFA. Individuals, foreign organizations and governments, nonprofit organizations exempt from taxation under section 501(c)(4) of the Internal Revenue Code that engage in lobbying, and "for-profit" organizations are not eligible. Nonprofit organizations, as defined by OMB Circular A-122, located at 2 CFR Part 230, means any corporation, trust, association, cooperative, or other organization which: (1) is operated primarily for scientific, educational, service, charitable, or similar purposes in the public interest; (2) is not organized primarily for profit; and (3) uses its net proceeds to maintain, improve, and/or expand its operations. Applicants must be eligible at the time of their submission.

Eligible Minority Academic Institutions, as described in Section I, are strongly encouraged to apply for funding under this competition.

Coalitions: Groups of two or more eligible applicants may choose to form a coalition and submit a single application under this RFA; however, one entity must be responsible for the grant. Coalitions must identify which eligible organization will be the recipient of the grant and which eligible organization(s) will be subawardees of the recipient. Subawards and subgrants must be consistent with the definitions of those terms in 40 CFR Sections 30.2(ff) and 31.3, respectively. The recipient must administer the grant, will be accountable to EPA for proper expenditure of the funds and reporting, and will be the point of contact for the coalition. As provided in 40 CFR Section 30.2(gg) and 40 CFR Section 31.3, subrecipients or subgrantees are accountable to the recipient or grantee for proper use of EPA funding.

Coalitions may not include for-profit organizations that will provide services or products to the successful applicant. For-profit organizations are not eligible for subawards. For-profit organizations are eligible to receive contracts. Any contracts for services or products funded with EPA financial assistance must be awarded under the competitive procurement procedures of 40 CFR Subparts 30 and 31. The regulations also contain limitations on consultant compensation. (Please see 40 CFR Sections 30.27(b) or 31.36(j), as applicable.) For additional information, please review the following Federal Register: http://edocket.access.gpo.gov/2004/pdf/04-7867.pdf .

Eligible Activities: Under this RFA, assistance is available to eligible applicants for planning, research, monitoring, outreach, and implementation of the GLRI and GLWQA. To be eligible for funding, proposed projects must protect, enhance and/or restore the Great Lakes, including projects impacting connecting waterways such as Lake St. Clair and the St. Lawrence River (at or upstream from the point at which the St. Lawrence River becomes the international boundary between Canada and the United States). Information about the GLRI can be found at http://www.epa.gov/greatlakes/glri. Applications for other activities will be rejected.

Ineligible Activities: If an application is submitted that includes any ineligible activities, including those listed below and in Section I, that portion of the application will be ineligible for funding and may, depending on the extent to which it affects the application, render the entire application ineligible. The following are ineligible activities:

- Traditional water or wastewater infrastructure projects (other than "green infrastructure"[6] projects that improve habitat and other ecosystem functions in the Great Lakes) that are eligible for funding from: 1) a state water pollution control revolving fund established under title VI of the Clean Water Act; or 2) a state drinking water revolving loan fund established under Section 1452 of the Safe Drinking Water Act (42 U.S.C. Section 300j–12).
- Any activities/projects that are specifically required by a draft or final NPDES permit or by a consent decree.
- Basic research, meaning research for the purpose of expanding knowledge or general understanding. This differs from "applied" research for the purpose of implementing solutions for actual problems. If a practical use of the research in question is less than 10 years away, then that research will be deemed to be applied research; however, if a practical use of the research in question is reasonably expected to be 10 or more years away, then that research will be deemed to be basic research.
- Land acquisition other than acquisition of easements.
- Removal or remediation of contaminated sediments.
- Projects the principal purpose of which is general operating support.
- Projects that EPA determines will be funded by EPA through a non-competitive state or tribal grant.
- Projects principally pertaining to restoration or remediation at Areas of Concern.
- Projects principally pertaining to habitat and wildlife protection and restoration.
- Projects principally pertaining to Asian carp.

In addition, the respective categories in Section I of the RFA describe certain activities that are ineligible activities for those categories (but could be eligible elsewhere), such as:

- Specified remedial actions, erosion control measures; and human health projects described in Section I.A (Toxic Substance Reduction).
- Monitoring activities described in Section I.C.2 (Watershed Remediation).

---

[6] Green Infrastructure includes a wide array of practices at multiple scales that manage and treat stormwater and that maintain and restore natural hydrology by infiltrating, evapotranspiring and capturing and using stormwater. On a regional scale, green infrastructure is the preservation and restoration of natural landscape features, such as forests, floodplains and wetlands, coupled with policies such as infill and redevelopment that reduce overall imperviousness in a watershed. On the local scale, green infrastructure consists of site- and neighborhood-specific practices, such as bioretention, trees, green roofs, porous pavements and cisterns.

Match or Cost-Share: There is no cost-sharing or matching requirement as a condition of eligibility under this RFA. However, see Section IV.C.2.b.iii and Section V for additional information regarding applicants who propose voluntary matches and additional funds/resources to support the project.

Threshold Eligibility Criteria: These are requirements that, if not met by the applicant by the time of application submission, will result in elimination of the application from consideration for funding. Only applications for eligible activities from eligible entities (see above) that meet these criteria by the time of application submission will be evaluated against the ranking factors in Section V of this RFA. Applicants deemed ineligible for funding consideration as a result of the threshold eligibility review will be notified within 15 calendar days of the ineligibility determination.

1. a. Applications must substantially comply with the application submission instructions and requirements set forth in Section IV of this RFA or else they will be rejected. Where a page limit is stated for the Narrative Proposal in Section IV, pages in excess of the limitation will not be reviewed.

   b. Applications must be submitted to EPA through http://www.grants.gov (or any approved alternate means as discussed in Section IV.B). Applicants are responsible for ensuring that their applications are submitted by the submission deadline identified in Section IV. Neither zipped file submissions nor faxed submissions will be accepted.

   c. An application submitted after the deadline will be considered late and rejected without further consideration unless the applicant can clearly demonstrate and document that its application was late due solely to EPA mishandling or technical problems attributable to grants.gov.

2. Applicants may submit more than one application under this RFA so long as each application is for a different project and is separately submitted.

3. Each application must address one, and only one, focus area, and one, and only one, category under a focus area, as described in Section I of the RFA.

4. Applications must meet any additional eligibility requirements described in Section I that apply to the respective category and focus area. Such additional eligibility requirements include:

   - Pollution Prevention and Toxics Reduction (Section I.A.) – Inclusion of necessary actions described in that section.
   - Watershed Remediation (Section I.C.2) – $1,000,000 maximum federal amount (applications requesting in excess of that will be rejected; implementation of applicable practices and measures; identification of applicable components; and estimation of applicable results).

- Increasing Technical Expertise to Accelerate Nutrient Reduction (Section I.C.3) – Accelerate applicable nutrient reduction and complement targeted efforts in the Maumee River, Fox River, and/or Saginaw River basins.

Applicants should contact the applicable individuals listed in Section VII of this RFA with any questions about the threshold eligibility requirements that may apply to a particular category.

IV. APPLICATION AND SUBMISSION INFORMATION

A. How to Obtain an Application Package:

Applicants can download individual grant application forms from EPA's Office of Grants and Debarment website at: http://www.epa.gov/ogd/AppKit/application.htm.

B. Mode of Application Submission:

Applicants, except in the limited circumstances noted below, must submit their applications electronically through http://www.grants.gov , and the application must be submitted by the applicant's authorized official representative as explained in the Appendix II grants.gov instructions. Applicants who do not have the technical capability to apply electronically through www.grants.gov must contact Michael Russ (312-886-4013 / RFA2012@glnpo.net to request alternate submission instructions. Any applications submitted through alternate means must still comply with all the requirements, instructions and deadlines in this RFA.

All applications must be prepared and include the information as described below in Section IV.C "Content of Application Package Submission." Applications must meet the submission requirements specified below and be received by the submission deadline.

For technical questions about electronic submittal of applications via http://www.grants.gov/ contact the Grants.gov Contact Center at 1-800-518-4726 or email Grants.gov at support@grants.gov. For calls from outside the United States, please dial 606-545-5035 to speak with a Contact Center representative. For questions regarding EPA's receipt of your application, see the general RFA contact information in Section VII of this RFA.

C. Content of Application Package Submission:

1. Necessary Grant Application Forms:

All application submissions must contain completed and signed grant application forms, as well as a Narrative Proposal, as described below. The grant application forms are

available at http://www.epa.gov/ogd/AppKit/application.htm and are listed in Appendix II.

2. Narrative Proposal:

Narrative Proposals (including the Summary Information Page, Workplan, and Detailed Budget Narrative) must be no more than twenty single-spaced pages in length and include the items below in the requested order**. Excess pages will not be reviewed.** Each Narrative Proposal must be formatted for 8½" x 11" paper and should use no smaller than an 11-point Times New Roman font with 1" margins. Readability is of paramount importance. Do not include more than one application in any file. Please do not zip the file or use a zip extension for your file because we will not be able to open it.

It is recommended that confidential business information not be included in your application.

a. **Summary Information Page (should not exceed one page):**

i. **Funding Opportunity Number, Focus Area, and Category.** The RFA number is EPA-R5-GL2012-1. Identify the focus area from Section I to which the proposal relates by choosing one from among "Toxic Substances and Areas of Concern," "Invasive Species," "Nearshore Health and Nonpoint Source Pollution," and "Accountability, Education, Monitoring, Evaluation, Communication, and Partnerships." Identify the category listed under a focus area to which the proposal relates. Identify only one focus area and one category per application submission.

ii. **Project Title.** Please limit to 60 characters, or EPA reserves the right to change the project title for its administrative convenience. Applicants who are submitting separate, complementary proposals may wish to use a proposal title with the same first words followed by a hyphen and a unique project title.

iii. **Applicant Information.** Include applicant (organization) name, address, contact person, phone number, fax, e-mail address, congressional district, and DUNS number (see Section VI.D). *Do not include private information.*

iv. **Type of Organization.** Specify, consistent with your SF 424, one of the following:

a) Local Government (includes County, Municipal, Inter-municipal organizations, Township, Special Purpose District and other entities defined as local governments at 40 CFR Section 31.3);

b) State Agency (includes Interstate);
c) Indian Tribe
d) College and University (includes private and state institutions);
e) Not for Profit/Non-profit
f) Other

v. **Proposed Funding Request.** The total dollar amount requested from EPA.

vi. **Project Duration Period.** Provide beginning and ending dates. (Except as specifically provided in Section I of the RFA, projects are anticipated to begin no earlier than July 1, 2012 and end no later than September 30, 2014.)

vii. **Brief Project Description.** Summarize the proposed project in a clear and succinct manner, including expected outputs, outcomes and environmental benefits resulting from implementation of the project. Include environmental KEY TERMS that could be used as search terms (*e.g.*, water quality, toxins, mercury, *etc*.). Do not use acronyms. Should the proposal be selected and a grant awarded, this description may be posted to the EPA Web, which has a 595 character limit, including spaces, to this field. EPA reserves the right to make unilateral changes to conform to posting requirements. See https://restore.glnpo.net/glas_pub/qareport.htm for examples.

viii. **Project Location.** Specify the project location, if applicable, including 8- or 12-digit HUC code (available from http://water.usgs.gov/wsc/reg/04.html); latitude and longitude; state; congressional district; county; city; and 5-digit ZIP Code.

b. Work Plan. The Work Plan for each proposed project must explicitly describe how the proposed project meets the guidelines established in Sections I-III of this RFA (including the threshold eligibility criteria in Section III) and must address each of the evaluation criteria set forth in Section V.

i. **Project Summary and Approach:** Describe with specificity the nature of the proposed project including what will be done, by whom, how, and when. Outline the steps to be taken and the significant milestones to be achieved to complete the proposed project as well as the estimated dates of these achievements, including the submittal of the final report. Include a statement of the project's relevance to the Great Lakes, particularly (1) the needs and priorities of the GLRI Action Plan (http://greatlakesrestoration.us/action/wp-content/uploads/glri_actionplan.pdf), or (2) Great Lakes protection and restoration pursuant to Sub-objective 2.2.4 (Improve the Health of Great Lakes ecosystems) of EPA's Strategic Plan http://www.epa.gov/ocfo/plan/plan.htm. (It is sufficient for the purpose of clause (ii) to include a general statement of how the project will protect and restore the

Great Lakes ecosystem without specifying a connection to the Great Lakes Index or remediation of contaminated sediments.**)** **If your proposed project is for a category or focus area that requires specific information described in Section I, include that information in this section.**

ii. **Results – Outputs and Outcomes:** Specify the quantitative and qualitative expected outputs and outcomes of the proposed project including but not limited to those specifically identified in Section I for the different categories, any other applicable objectives or measures from the GLRI Action Plan, and the approach and measurements that will be used to track and measure your progress towards achieving the applicable outputs and outcomes. Specify the estimated quantifiable environmental and economic outputs, outcomes, and results of the proposed project, including affected pollutants, industry sectors, economic impacts, habitats, and/or species. Include an estimate of, *e.g.*, the amount of chemicals to be removed, the amount of discharges of toxic chemical substances that will be prevented, acres protected or restored, *etc*. Describe the anticipated accuracy of that estimate, including applicable limitations. In addition, provide a timetable or schedule with target dates projected for major tasks, accomplishments and deliverables.

iii. **Collaboration, Partnerships, and Overarching Plans:** Describe your approach for promoting and/or obtaining collaboration and support from the public and private sectors in performing the project in order to expand its impact, and also describe whether and how your project complements other projects or activities being performed to achieve a larger beneficial impact from your project. List the proposed groups that will be involved in the project and any related projects and studies, and what each of the groups' roles will be in the project's staffing, funding, design and implementation. Describe the type of collaboration/support proposed, how you will ensure that it will materialize during project performance, and what role it will play in the overall project. (Any letters demonstrating evidence of collaboration and support from the public or private sector should be attached as part of item 11 of the Application Materials listed in Appendix II.) Describe how you will coordinate activities of the project with related or complementary projects and studies. PLEASE CAREFULLY REVIEW SECTION IV. I (Questions and Answers Pertaining to Contracts and Subawards) IF YOU INTEND TO PROVIDE EPA FUNDS TO ANY COLLABORATING ORGANIZATION.

If the project is part of any applicable overarching plan for protection and restoration of an important Great Lakes place-based effort (*e.g.*, LaMPs, RAPs, state Great Lakes plans, TMDLs and watershed management plans) describe the purpose and effect of the project in such plan. (Such plans may cross over one or more focus areas and categories of activities. See Appendix I for a statement of LaMP priorities, which may be applicable to each of the respective focus areas and activity categories.) Provide an active Internet link (URL) for that

overarching plan or, if a URL is not available, attach the plan as part of item 13 of the Application Materials listed in Appendix II. For nearshore and nonpoint source pollution projects which are part of an overarching, comprehensive plan, describe the targeted restoration potential, including the extent to which: 1) identifiable impairments have been identified and the causes for those impairments have been clearly established; 2) solutions to remediation of the impairments have been identified; 3) broadly supported implementation activities have been designed; and 4) there is potential for significant measurable results. (Such extent shall be considered as part of the scoring under the Project Summary and Approach criterion in Section V.A.1.)

In addition, for projects submitted under the Category I.C.2 (Watershed Remediation), applicants may propose a voluntary cost-match to provide additional support for the project and this cost-match may specifically be considered during the selection process for these projects as described in Section V. Applicants proposing a voluntary cost-match for these projects should describe the voluntary cost-match, how they will ensure it is provided during project performance, and what role it will play in the overall project. If EPA accepts an offer for a voluntary cost-match, applicants must meet the matching commitment as a condition of receiving EPA funding. The recipient is legally-obligated to meet any proposed voluntary cost-match that is included in the approved project budget because the grant agreement will include the voluntary cost-match. Applicants may use their own funds or other resources for a voluntary cost-match if the standards at 40 CFR Sections 30.23 or 31.24, as applicable, are met. Only eligible and allowable costs may be used for voluntary cost-match. Other federal grants may not be used as voluntary cost-matches without specific statutory authority (*e.g.,* HUD's Community Development Block Grants).

For projects submitted under the other categories identified in this RFA, if the applicant is proposing to provide a voluntary cost-match or other form of additional funds/resources to demonstrate support for the project, they should describe that in this section of the proposal. They should also describe how they will ensure that the voluntary cost-match or other funds/resources will be provided during project performance, and what role they will play in the overall project. Any additional funds/resources, including voluntary cost-matches and their source, must be identified in the application and, if applicable, on appropriate grant application forms. The additional funds or other resources need not be for eligible and allowable project costs under the EPA assistance agreement unless the applicant proposes to provide a voluntary cost-match. If EPA accepts an offer for a voluntary cost-match, applicants must meet their sharing commitment as a condition of receiving EPA funding. The recipient is legally-obligated to meet any proposed voluntary cost-match that is included in the approved project budget because the grant agreement will include the voluntary cost-match. Applicants may use their own funds or other resources for

a voluntary cost-match if the standards at 40 CFR Sections 30.23 or 31.24, as applicable, are met. Only eligible and allowable costs may be used for voluntary cost-matches. Other federal grants may not be used as voluntary cost-matches without specific statutory authority (*e.g.*, HUD's Community Development Block Grants).

Any voluntary cost-match should also be identified below in the Budget section (E). If subawards or subcontracts are involved, please also refer to the discussion of those topics in Section IV.I below.

iv. **Community-Based Focus and Environmental Justice Impacts:** Demonstrate how the proposed project will address the needs and concerns of local communities and other interested parties, groups, or populations that are, or have been, affected by the environmental and/or other issues (including any adverse environmental impacts that have disproportionately affected them) that the project is intended to address[7]. Demonstrate how the applicant will engage and work with community-based organizations (as defined in this RFA) and other appropriate parties to enhance project effectiveness and/or efficiency, including the applicant's plans for making subawards, as necessary and appropriate (see Section IV.I) to community-based organizations to enhance project effectiveness and/or efficiency. Applicants, not EPA, will select their subawardees and the applicant must demonstrate in its proposal that the community-based organization(s) are willing to accept the subaward and have the capacity to effectively administer and perform the agreement. Selected applicants who propose to make subawards, including those to community-based groups, must follow proper procedures in making subawards and will be expected to make the subawards consistent with their application. The award will include a term and condition requiring the applicant to make the subawards consistent with their application.

v. **Programmatic Capability and Past Performance:** Submit a list (of no more than 5) of federally-funded assistance agreements[8] (including previous GLRI awards from EPA or other federal sources) similar in size, scope and relevance to the proposed project that the applicant has previously been awarded and describe: (1) whether, and how, you were able to successfully complete and manage those agreements; (2) your history of meeting the reporting requirements under those agreements, including whether you adequately and timely reported on your progress towards achieving the expected outputs and outcomes of those agreements (and if not, explain why not); and (3) whether you submitted acceptable final technical reports under the agreements. In evaluating applicants under these factors in Section V, EPA will consider the information provided by the applicant and may also consider relevant information from other sources,

---

[7] See the footnote in Section V.A.4 for factors potentially indicating disproportionate impacts.

[8] Assistance agreements include federal grants and cooperative agreements, but not federal or other contracts.

including information from EPA files and from current and prior federal agency grantors (*e.g.*, to verify and/or supplement the information provided by the applicant).

Please Note: If you received a GLRI award in 2010 and/or 2011, you should list the award(s) and provide the information described above. In addition, for these awards please provide an explanation of and documentation supporting your quarterly rate of expenditure on those prior GLRI projects up through the date of the applicant's submission.

If you do not have any relevant or available past performance or reporting information, please indicate this in the proposal and you will receive a neutral score for these factors (a neutral score is half of the total points available in a subset of possible points). Failure to indicate this may result in 0 points for these factors.

In addition, provide information on your organizational experience and your plan for timely and successfully achieving the objectives of the proposed project, and your staff expertise/qualifications, staff knowledge, and resources (or the ability to obtain them) to successfully achieve the goals of the proposed project.

vi. **Job Creation.** Describe how the project is expected to promote and/or facilitate job creation consistent with the application review criteria in Section V.A.6.

vii. **Education/Outreach.** Describe how project results will be disseminated to interested stakeholders; your demonstrated track record of outreach to citizens on environmental issues; and the potential of the project for transferability and applicability to other places in accordance with the application review criteria in Section V.A.7.

c. **Detailed Budget Narrative: (Also see Appendix III, Budget Sample).** Clearly explain how EPA funds will be used. Use this section to provide a narrative description of the budget found in the SF-424A. Applicants must itemize costs related to personnel, fringe benefits, contractual costs, travel, equipment, supplies, other direct costs, indirect costs, and total costs. Applicants should use whole dollar amounts.

i. **Management Fees:** When formulating budgets for applications, applicants must not include management fees or similar charges in excess of the direct costs and indirect costs at the rate approved by the applicant's cognizant audit agency, or at the rate provided for by the terms of the agreement negotiated with EPA. The term "management fees or similar charges" refers to expenses added to the direct costs in order to accumulate and reserve funds for ongoing business expenses, unforeseen liabilities, or for other similar costs that are not allowable under EPA

assistance agreements. Management fees or similar charges may not be used to improve or expand the project funded under this agreement, except to the extent authorized as a direct cost of carrying out the scope of work.

ii. **Expeditious Spending and Sufficient Progress in the use of GLRI Funds:** Applicants should explain how, if they are awarded a grant, they will ensure that the funding will be used expeditiously. Include expenditure projections, with quarterly fiscal projections and milestones, for the life of the grant.

3. **Other Attachments.** The additional attachments listed in Appendix II are not part of the Narrative Proposal and are not included in the 20 page limit; however, they may, as appropriate, be considered during evaluations. For additional information about each of these attachments, see the descriptions contained in Appendix II.

D. **Submission:** Eligible applicants must submit applications through http://www.grants.gov per the instructions in Appendix II or through any approved alternate method as discussed above in Section IV.B.

E. **Submission Deadline:** Applications must be submitted to EPA through http://www.grants.gov by 11:59 p.m. Eastern Daylight Time, on May 24, 2012.

F. **Notification:** Within two weeks after the due date, EPA intends to post a link to project information (including title and identification number) to: http://epa.gov/greatlakes/fund/2012rfa01. ALL APPLICANTS SHOULD CHECK THIS POSTING TO VERIFY THAT THEIR SUBMISSIONS HAVE BEEN INCLUDED IN EPA'S DATABASE. See Section VII for contact information if you do not receive a confirmation or if your project is not posted. All Applicants will be contacted following selections to tell them whether or not they have been selected. Selection information will also be posted to a page linked to: http://epa.gov/greatlakes/fund/2012rfa01.

G. **Confidentiality:** EPA recommends that you do not include confidential business information ("CBI") in your proposal/application. However, if CBI is included, it will be treated in accordance with 40 CFR Section 2.203. Applicants must clearly indicate which portion(s) of their proposal/application they are claiming as CBI. EPA will evaluate such claims in accordance with 40 CFR Part 2. If no claim of confidentiality is made, EPA is not required to make the inquiry to the applicant otherwise required by 40 CFR Section 2.204(c)(2) prior to disclosure. The Agency protects competitive proposals/applications from disclosure under applicable provisions of the Freedom of Information Act prior to the completion of the competitive selection process.

Applicants should be aware that under Public Law No. 105-277, data produced under an award is subject to the Freedom of Information Act.

H. **Communications with Applicants:** In accordance with EPA's Assistance Agreement Competition Policy (EPA Order 5700.5A1), EPA staff will not meet with individual

applicants to discuss draft applications, provide informal comments on draft applications, or provide advice to applicants on how to respond to ranking criteria. Applicants are solely responsible for the contents of their applications. However, consistent with the provisions in the announcement, EPA will respond to questions from individual applicants regarding threshold eligibility criteria, administrative issues related to the submission of the proposal, and requests for clarification about the announcement. In addition, if necessary, EPA may clarify threshold eligibility issues with applicants prior to making an eligibility determination. Submit questions using the form available from http://epa.gov/greatlakes/fund/2012rfa01. EPA will respond to questions received through May 10, 2012, but cannot guarantee that it will respond to questions received thereafter.

EPA will also host two webinars during which EPA will discuss this RFA and respond to questions. Since all questions may not be able to be asked and answered during the scheduled times for the call, questions should be submitted in advance using the form available from http://epa.gov/greatlakes/fund/2012rfa01 mentioned above. The webinars will be broadcast live and will be archived for future playback. Pre-registration will be required for the webinars.

Webinar Schedule:

Dates: Thursday, May 3, 2012 at 2 p.m. Central Daylight Time
Monday, May 14, 2012 at 10:00 a.m. Central Daylight Time

Topic: EPA Great Lakes Restoration Initiative Request for Applications

Further details, including a link for the Webinar, will be available from:
http://epa.gov/greatlakes/fund/2012rfa01

I. Questions and Answers Pertaining to Contracts and Subawards:

1. Can funding be used for the applicant to make subawards, acquire contract services, or fund partnerships?

EPA awards funds to one eligible applicant as the recipient even if other eligible applicants are named as partners or co-applicants or members of a coalition or consortium. The recipient is accountable to EPA for the proper expenditure of funds.

Funding may be used to provide subgrants or subawards of financial assistance, which includes using subawards or subgrants to fund partnerships, provided the recipient complies with applicable requirements for subawards or subgrants including those contained in 40 CFR Parts 30 or 31, as appropriate. Applicants must compete contracts for services and products, including consultant contracts, and conduct cost and price analyses, to the extent required by the procurement provisions of the regulations at 40 CFR Parts 30 or 31, as appropriate. The regulations also contain limitations on consultant compensation.

Applicants are not required to identify subawardees/subgrantees and/or contractors (including consultants) in their application. However, if they do, the fact that an applicant selected for award has named a specific subawardee/subgrantee, contractor, or consultant in the application EPA selects for funding does not relieve the applicant of its obligations to comply with subaward/subgrant and/or competitive procurement requirements as appropriate. Please note that applicants may not award sole source contracts to consulting, engineering or other firms assisting applicants with the application solely based on the firm's role in preparing the application.

Successful applicants cannot use subgrants or subawards to avoid requirements in EPA grant regulations for competitive procurement by using these instruments to acquire commercial services or products from for-profit organizations to carry out its assistance agreement. The nature of the transaction between the recipient and the subawardee or subgrantee must be consistent with the standards for distinguishing between vendor transactions and subrecipient assistance under Subpart B Section 210 of OMB Circular A-133, and the definitions of subaward at 40 CFR Section 30.2(ff) or subgrant at 40 CFR Section 31.3, as applicable. EPA will not be a party to these transactions. Applicants acquiring commercial goods or services must comply with the competitive procurement standards in 40 CFR Part 30 or 40 CFR Part 31.36 and cannot use a subaward/subgrant as the funding mechanism.

2. How will an applicant's proposed subawardees/subgrantees and contractors be considered during the evaluation process described in Section V of the RFA?

Section V of the RFA describes the evaluation criteria and evaluation process that will be used by EPA to make selections under this RFA. During this evaluation, except for those criteria that relate to the applicant's own qualifications, past performance, and reporting history, the review panel will consider, as appropriate and relevant, the qualifications, expertise, and experience of:

(i) an applicant's named subawardees/subgrantees identified in the application if the applicant demonstrates in the application that if it receives an award that the subaward/subgrant will be properly awarded consistent with the applicable regulations in 40 CFR Parts 30 or 31. For example, applicants must not use subawards/subgrants to obtain commercial services or products from for profit firms or individual consultants.

(ii) an applicant's named contractors, including consultants, identified in the application if the applicant demonstrates in its application that the contractors were selected in compliance with the competitive procurement standards in 40 CFR Part 30 or 40 CFR Section 31.36, as appropriate. For example, an applicant must demonstrate that it selected the contractors competitively or that a proper non-competitive sole-source award consistent with the regulations will be made to the contractors, that efforts were made to provide small and disadvantaged businesses with opportunities to compete, and that some form of cost- or price-analysis was conducted. EPA may not accept sole-source justifications for contracts for services or products that are otherwise readily available in the commercial marketplace.

EPA will not consider the qualifications, experience, and expertise of named subawardees/subgrantees and/or named contractors during the application evaluation process unless the applicant complies with these requirements.

J. Intergovernmental Review: Executive Order 12372, Intergovernmental Review of Federal Programs, may be applicable to awards resulting from this announcement. Applicants selected for funding may be required to provide a copy of their application to their State Point of Contact (SPOC) for review, pursuant to Executive Order 12372. This review is not required before submitting an application and not all states require such a review. A listing of State Point of Contacts (SPOC) may be viewed at: www.whitehouse.gov/omb/grants/spoc.html

V. APPLICATION REVIEW AND SELECTION PROCESS

A. Application Review:

Applications meeting the threshold eligibility criteria in Section III will be evaluated based on the criteria set forth below. Applicants should directly and explicitly address these criteria as part of their Narrative Proposal and application submission. Each submittal will be rated under a point system, with a total of 100 points possible. Applicants will be evaluated based on the quality and extent to which they address the criteria; the failure to provide applicable information in the application may affect the score assigned for a criterion.

1. **Project Summary and Approach - 15 points:** Applicants will be evaluated based on their strategic and technical approach for performing the project including the soundness and logic of these approaches. Results of any applicable scientific peer review submitted by the applicant may be considered as a part of the evaluation under this criterion.

   Applicants may score higher on this criterion to the extent they demonstrate in their Narrative Proposal one or more of the following elements:

   - A clear, rather than a weak, connection to protection and restoration of the Great Lakes themselves;
   - Immediacy and timeliness in project implementation ("shovel ready") and in attaining proposed and expected outputs and outcomes including protection, enhancement and restoration;
   - Consideration of climate change impacts through vulnerability assessments and/or the integration of climate change adaptation measures into their project.

   A project submitted pursuant to Section I.A (Toxic Substance Reduction) of this RFA for a reduction in the use of mercury in products, better management of mercury-containing wastes, or reduction in mercury emissions may receive a higher score on this criterion to the extent the project helps states, Indian tribes, and local governments implement the recommendations of the Great Lakes Regional Collaboration's *Mercury in Product Phase-down Strategy* (http://glrc.us/documents/MercuryPhaseDownStrategy06-19-2008.pdf) and/or the Great Lakes Regional Collaboration's *Mercury Emissions Strategy* (http://glrc.us/documents/MercuryEmissionsPhaseDownStrategy12-07-2010.pdf).

   A project submitted pursuant to Section I.C (Nearshore Health and Nonpoint Source Pollution) of this RFA, may receive a higher score on this criterion to the extent that: identifiable impairments have been identified and the causes for those impairments have been clearly established; solutions to remediation of the impairments have been identified; broadly supported implementation activities have been designed; and the project demonstrates the potential for significant measurable results.

   A project submitted pursuant to Section I.C.2 (Watershed Remediation), which is located in the geographic areas targeted in the GLRI Action Plan (Fox River, Saginaw River, Maumee

River, St. Louis River, and Genesee River) may receive a higher score on this criterion; and watershed remediation projects located in the priority sub-watersheds of the Maumee River (*i.e.,* Upper Blanchard River Watershed), the Lower Fox River (*i.e.,* Upper East River and Upper Duck Creek), and the Saginaw River (*i.e.,* Swartz and Kearsley Creek Watersheds) may also receive a higher score on this criterion.

A project submitted pursuant to Section I.C.3 (Increasing Technical Expertise to Accelerate Nutrient Reduction) of this RFA may receive a higher score on this criterion if it is located in the priority subwatersheds of the Maumee River (*i.e.,* Upper Blanchard River Watershed), the Lower Fox River (*i.e.,* Upper East River and Upper Duck Creek), or the Saginaw River (*i.e.,* Swartz and Kearsley Creek Watersheds).

A project submitted pursuant to Section I.D.1 (Increasing Climate Change Resiliency in Great Lakes Communities) of this RFA may receive a higher score on this criterion to the extent it is designed to accomplish one or more of the items (*i.e.,* vulnerability identification; risk demonstration; planning, development, and prioritization of actions; and implementation of actions) identified in that Section.

2. **Outputs and Outcomes – 20 points:**

   (a) Applicants will be evaluated based on their approach for demonstrating how they will achieve the expected and proposed project outputs and outcomes applicable to the focus area and category to which the application relates including but not limited to those identified in Section I for the applicable category. **(15 points)**

   (b) Applicants will be evaluated based on their plan and approach for measuring and tracking their progress towards achieving the expected and proposed project outputs and outcomes including but not limited to those identified in Section I that apply to the applicable category to which the application relates. **(5 points)**

3. **Collaboration, Partnerships, and Overarching Plans (see Section IV.C.2.b.iii.) – 10 points:** Applicants will be evaluated based on the extent to which they demonstrate that they will work in partnership with appropriate partners (such as governmental agencies, applicable regulatory entities, community groups, businesses, stakeholders, Indian tribes, states, cities, and counties) to effectively and efficiently implement the proposed project and whether their project is coordinated with and/or complements other projects or activities being performed by the applicant or others which will result in a greater positive environmental impact from the applicant's project. This includes evaluating the applicant's plans for obtaining collaboration and support from the public and private sectors in performing the project in order to expand its impact, the scope of the partnership (including the diversity and number of partners), the type of collaboration proposed, the likelihood that it will materialize during project performance, and what role it will play in the overall project. An example of a collaboration or partnership that would be considered under this criterion would be a proposal by an applicant (a single entity) to use GLRI funding to make

subawards to one or more identified governmental or nonprofit subrecipients that would work collaboratively with the applicant implement an overarching plan.

Applicants may score higher on this criterion to the extent they demonstrate in their Narrative Proposal the protection or restoration potential of the project as part of an overarching, comprehensive plan (including Lakewide Management Plans, Remedial Action Plans, state Great Lakes plans, watershed plans (including those which impact Areas of Concern), and other place-based plans including TMDLs.

4. **Community-Based Focus and Environmental Justice Impacts- 10 points:** Under this criterion, applicants will be evaluated based on the quality and extent to which their proposal demonstrates how the proposed project will address the needs and concerns of local communities and other interested parties, groups, or populations that are, or have been, affected by the environmental and/or other issues (including any adverse environmental impacts that have disproportionately affected them) that the project is intended to address[9]. This includes evaluating the quality and extent to which the applicant demonstrates: 1) how it will engage and work with community-based organizations (as defined in this RFA) and other appropriate parties to enhance the effectiveness and/or efficiency of the project; and 2) the applicant's plans for making subawards, as necessary and appropriate (see Section IV.I) to incorporated community-based organizations to enhance project effectiveness and/or efficiency. Applicants, not EPA, will select their subawardees and the applicant must demonstrate in its proposal that the community-based organization(s) are willing to accept the subaward and have the capacity to effectively administer and perform the agreement. Selected applicants who propose to make subawards, including awards to community-based organizations, must follow proper procedures in making subawards and will be expected to make the subawards consistent with their proposal. The award will include a term and condition requiring the applicant to make the subawards consistent with their proposal.

5. **Programmatic Capability and Past Performance – 20 points.** Under this criterion, applicants will be evaluated based on their ability to successfully complete and manage the proposed project taking into account their:

   a. past performance in successfully completing and managing the assistance agreements identified in response to Section IV.C.2.b.v of the RFA **(7 points)**;
   b. history of meeting the reporting requirements under the assistance agreements identified in response to Section IV.C.2.b.v of the RFA including: 1) whether the applicant submitted acceptable final technical reports under those agreements and;

[9] Factors potentially indicating disproportionate impacts include: differential proximity and exposure to environmental hazards; greater susceptibility to adverse effects from environmental hazards (due to causes such as genetic predisposition, age, chronic medical conditions, lack of health care access, or poor nutrition); unique environmental exposures because of practices linked to cultural background or socioeconomic status (for example, subsistence fishing or farming); cumulative effects from multiple stressors; reduced ability to effectively participate in decision-making processes (due to causes such as language barriers, inability to access traditional communication channels, or limited capacity to access technical and legal resources); and degraded physical infrastructure, such as poor housing, poorly maintained public buildings (*e.g.*, schools), or lack of access to transportation.

2) the extent to which the applicant adequately and timely reported on their progress towards achieving the expected outputs and outcomes under those agreements and in the event such progress was not being made, whether the applicant adequately reported on and explained the lack of progress **(3 points)**;

c. organizational experience and plan for timely and successfully achieving project objectives **(3 points)**, and

d. staff expertise/qualifications, staff knowledge, and resources (or the ability to obtain qualified staff and resources on a timely basis) to successfully achieve project goals (**7 points)**.

In evaluating applicants under items (a) and (b) of this criterion, EPA will consider the information provided by the applicant and may also consider relevant information from other sources including agency files and prior/current grantors (*e.g.*, to verify and/or supplement the information supplied by the applicant). If the applicant does not have any relevant or available past performance or reporting information, this should be indicated in the Narrative Proposal and the applicant will receive a neutral score for these sub-factors (a neutral score is one half of the points available for the item). If the applicant does not provide any response for these items, it may receive a score of 0 for these factors.

[NOTE: Points may be reduced from an applicant's score under item a, above, if it has previously been awarded GLRI funds and such funds, or a significant portion of them, have not been expended expeditiously as of the date of the applicant's submission without adequate explanation. Applicants must provide an explanation if they have failed to expeditiously expend previously awarded GLRI funds or a significant portion thereof.]

[NOTE: Points may be reduced from an applicant's score if the applicant, without adequate explanation, has not demonstrated an ability to timely comply with current American National Standard Specifications and Guidelines for Quality Systems for Environmental Data Collection and Environmental Technology Programs, ANSI/ASQC E4-1994.]

6. **Job Creation – 5 points.** Applications will be evaluated based on the extent to which the applicant can demonstrate how the proposed project will effectively facilitate and/or promote job creation as part of effective and efficient project implementation. The application may receive a higher score under this criterion to the extent the applicant can sufficiently and credibly demonstrate the potential for the project to create numerous permanent jobs, including jobs created on a civilian conservation corps model for environmental restoration projects.

7. **Education/Outreach – 5 points:** Applicants will be evaluated based on the effectiveness of their education/outreach plans to disseminate project results to interested stakeholders including, but not limited to, whether the applicant has a demonstrated track record of

outreach to inform citizens on environmental issues and the potential of the project for transferability and applicability to other places.

8. **Budget – 15 points:** Applications will be evaluated based on the reasonableness, necessity and allowability (of costs) of the proposed budget for the level of work proposed and for the expected benefits to be achieved. Applicants will also be evaluated on their plan for how, if they are awarded a grant, they will ensure that the funding will be used expeditiously.

   An applicant's budget and budget narrative must account for both federal funds and any non-federal funds (*e.g.*, any voluntary cost-share/match if applicable). Applicants must precisely describe in their budget narrative how they will account for any voluntary cost-share/match or other non-EPA funds and what role EPA funding will play in the overall project.

B. Selection Process:

1. **Evaluation:**

Applications will first be evaluated against the threshold factors listed in Section III. Only those applications which meet all of the threshold factors will be evaluated using the evaluation criteria listed above. Eligible applications will be evaluated based on the criteria above by review panels composed of federal agency staff or other appropriate reviewers. There will be separate review panels for evaluating eligible applications submitted under each category described in Section I. Review panels will rank the applications based on the criteria above and develop preliminary funding recommendations for presentation to the selection official(s). Information pertaining to proposed recommendations may be shared with appropriate governmental departments or agencies before selections are made in order to determine whether potential selections (a) are expected to be funded by another funding department or agency under the Initiative or any other known funding source or (b) could be affected by permitting or regulatory issues.

In reviewing applications submitted for projects under the Category I.C.2 (Watershed Remediation), the review panel will also consider any voluntary cost-match proposed by the applicant in terms of what the overall project will achieve (see Section IV.C.2.b.iii) and provide its assessment of the proposed voluntary cost-match to the selection official(s) described below who may consider it in making final selections for projects under this category.

Final funding decisions will be made by the selection official(s). In making the final funding decisions, the selection official(s) will consider the review panel rankings and recommendations and may also consider the following factors: any duplicate funding issues or permitting or regulatory issues as discussed above; program priorities; funding availability; appropriate balances of geographic and jurisdictional distribution of projects (*e.g.*, appropriate geographic distribution can include balancing projects among LaMPs to address each of their priorities); and organizational diversity in terms of applicant type selected to receive awards (*e.g.*, local government, state agency, Indian Tribe**,** college and university, or other not for profit entity) in order to help ensure a broad representation of

entity types receiving awards to promote program effectiveness. For projects submitted under the Category I.C.2 – Watershed Remediation – the selection official(s) may also consider the review panel's assessment of any proposed voluntary cost-match as discussed above.

Any final selections made out of rank order must be documented and justified based on the factors listed above. Once selections have been made, a funding recommendation will be developed and forwarded to the EPA approving official.

2. Conflict of Interest:

All reviewers will be required to sign a disclosure of conflict of interest statement and will be removed from the review of applications where an actual or potential conflict of interest (that cannot be mitigated) exists. The selection official(s) will also sign a conflict of interest statement.

VI. AWARD ADMINISTRATION

A. Award Notices and Status: Following evaluation of applications, all applicants will be notified regarding their status, as follows:

> EPA anticipates notification to *unsuccessful* applicants will be made via email or postal mail.
>
> EPA anticipates that notification to *finalists* will be made via email. The notification will advise them that their proposed project has been evaluated and forwarded to the EPA approving official for further consideration and possible award. This notification, which advises finalists that their proposed project has been forwarded to the approving official, **is not and should not be considered as** an authorization to begin performance. The award notice signed by the EPA award official is the authorizing document and will be provided through postal mail. This process is generally expected to take 60 to 90 days from the date of recommendation.

B. Administrative and National Policy Requirement: Successful applicants will be required to adhere to federal grants requirements, particularly those found in applicable OMB circulars on Cost Principles (A-21, A-87, or A-122), Administrative Requirements (A-102 or 110), and Audit Requirements (A-133) available from http://www.whitehouse.gov/omb/grants. This includes government-wide requirements pertaining to accounting standards, lobbying, minority or woman business enterprise, publication, meetings, construction, and disposition of property. EPA regulations governing assistance programs and recipients are codified in Title 40 of the Code of Federal Regulations. A listing and description of general EPA regulations applicable to the award of assistance agreements may be viewed at: http://www.epa.gov/ogd/AppKit/applicable_epa_regulations_and_description.htm.

Those requirements, GLNPO-specific requirements currently in effect, and the application materials that will be needed by applicants can also be found online at: http://www.epa.gov/greatlakes/fund/projreqs.html and http://www.epa.gov/greatlakes/fund/appforms.html.

C. Quality System Documentation: Grants involving the use or collection of environmental data will be required to develop quality system documentation. This documentation is required to be delivered to EPA within 90 days of award **and approved before grantees commence any data collection activities**. Applicants should build this requirement into their schedules and budget. A significant percentage of the grants awarded in 2010 and 2011 required quality system documentation. For specific guidance on GLNPO's quality requirements please see www.epa.gov/glnpo/qmp/ .

D. Central Contractor Registration (CCR) and Data Universal Numbering System (DUNS) Requirements: Unless exempt from these requirements under OMB guidance at 2 CFR Part 25 (*e.g.*, individuals), each applicant must:

1. Be registered in the CCR prior to submitting an application under this announcement. CCR information can be found at https://www.bpn.gov/ccr/ ;

2. Maintain an active CCR registration with current information at all times during which it has an active federal award or an application under consideration by an agency; and

3. Provide its DUNS number in each application it submits to the agency. Applicants can receive a DUNS number, at no cost, by calling the dedicated toll-free DUNS Number request line at 1-866-705-5711, or visiting the D&B website at: http://www.dnb.com .

If an applicant fails to comply with these requirements, it will, should the applicant be selected for award, affect their ability to receive the award.

E. Reporting Requirements: Applicants selected for funding shall provide quarterly narrative technical progress reports addressing financial and work progress and to input data directly into the online Great Lakes Accountability System database (see http://restore.glnpo.net/glas_pub/qareport.htm) which was developed for the purpose of collecting and reporting information about GLRI. (We note that EPA has estimated the annual burden associated with GLAS-related reporting at 41 hours and $2,600 per project, including time for reviewing instructions, searching existing data sources, gathering and maintaining the data needed, and completing and reviewing the information.) Special conditions requiring quarterly and semi-annual financial and progress reporting and a detailed final technical report, will be added to awards. GLRI reporting requirements are updated regularly and may be changed by the time of the actual award. These requirements can be found at http://www.epa.gov/greatlakes/fund/applicationpac/Help/HI4-Regs-Instructions-9-20-2010.pdf. Applicants should make provision for their expected reporting costs in the budget for their applications.

PLEASE NOTE: If selected, applicants may be asked to revise their anticipated fiscal expenditure projections on a quarterly basis in order to monitor the progress of the awarded project. These projections should be submitted as a part of the fiscal and technical reporting.

F. Negotiating Fair Share Objectives/Goals and Disadvantaged Business Enterprises (DBE) Annual Reporting: If selected, the requirements of 40 CFR Part 33 apply (see: http://ecfr.gpoaccess.gov - Participation by disadvantaged business enterprises in United States Environmental Protection Agency Programs). Applicants who do not already have negotiated DBE goals in place will be required to negotiate DBE fair share objectives/goals with the Region 5 DBE Coordinator. The recipient will be required to submit proposed DBE objectives/goals based on an availability analysis, or disparity study, of qualified DBE in their relevant geographic buying market for construction, services, supplies and equipment. In addition, recipients will be required to submit annual DBE Reports to the DBE Coordinator. Whenever procuring construction, equipment, services and supplies under an EPA financial assistance agreement, the recipient of GLRI funds must undertake good faith efforts to, and ensure that subrecipients, loan recipients, and prime contractors undertake good faith efforts to:

(1) Ensure that DBEs are made aware of contracting opportunities to the fullest extent practicable through outreach and recruitment activities. For tribal, state and local government recipients, this will include placing DBEs on solicitation lists and soliciting them whenever they are potential sources.

(2) Make information on forthcoming opportunities available to DBEs and arrange time frames for contracts and establish delivery schedules, where the requirements permit, in a way that encourages and facilitates participation by DBEs in the competitive process. This includes, whenever possible, posting solicitations for bids or proposals for a minimum of 30 calendar days before the bid or proposal closing date.

(3) Consider in the contracting process whether firms competing for large contracts could subcontract with DBEs. For tribal, state and local government recipients, this will include dividing total requirements when economically feasible into smaller tasks or quantities to permit maximum participation by DBEs in the competitive process.

(4) Encourage contracting with a consortium of DBEs when a contract is too large for one of these firms to handle individually.

(5) Use the services and assistance of the SBA and the Minority Business Development Agency of the Department of Commerce.

(6) If the prime contractor awards subcontracts, require the prime contractor to take the steps in paragraphs (1) through (5) of this section.

Records documenting compliance with the required good faith efforts shall be retained.

G. Exchange Network: EPA, states, territories, and Indian tribes are working together to develop the National Environmental Information Exchange Network, a secure, internet- and standards-based way to support electronic data reporting, sharing, and integration of both regulatory and non-regulatory environmental data.

States, Indian tribes and territories exchanging data with each other or with EPA should make the Exchange Network and the Agency's connection to it, the Central Data Exchange (CDX), the standard way in which they exchange data and should phase out any legacy methods they have been using.

More information on the Exchange Network is available at http://www.exchangenetwork.net.

H. Disputes: Assistance agreement competition-related disputes will be resolved in accordance with the dispute resolution procedures published in 70 FR (Federal Register) 3629, 3630 (January 26, 2005) which can be found at http://www.epa.gov/ogd/competition/resolution.htm. Copies of these procedures may also be requested by contacting russ.michael@epa.gov.

I. Non-profit Administrative Capability and Mandatory Training: Nonprofit applicants that are recommended for funding under this announcement are subject to pre-award administrative capability reviews consistent with Section 8b, 8c and 9d of EPA Order 5700.8 - Policy on Assessing Capabilities of Non-Profit Applicants for Managing Assistance Awards (http://www.epa.gov/ogd/grants/award/5700_8.pdf). In addition, nonprofit applicants that qualify for funding may, depending on the size of the award, be required to fill out and submit to EPA's Grants Management Office the *Administrative Capabilities Form*, with supporting documents, contained in Appendix A of EPA Order 5700.8.

In addition, EPA requires nonprofit recipients to take a course designed to help them understand assistance agreement regulations, the application process, management of their assistance agreements and the close-out process. Completion of this class is mandatory for all non-profit award recipients. Certification will be good for 3 years. See Mandatory Grant's Management Training for Non-Profit Applicants and Recipients, available from: http://www.epa.gov/ogd/.

J. Subaward and Executive Compensation Reporting: Applicants must ensure that they have the necessary processes and systems in place to comply with the subaward and executive total compensation reporting requirements established under OMB guidance at 2 CFR Part 170, unless they qualify for an exception from the requirements, should they be selected for funding.

For additional information, please see 2 CFR Part 170, Reporting Subaward and Executive Compensation Information (http://ecfr.gpoaccess.gov) and Additional Guidance on Subaward and Executive Compensation on Reporting (http://www.fsrs.gov).

K. Use of Grant Funds: An applicant that receives an award under this announcement is expected to manage assistance agreement funds efficiently and effectively and make sufficient

progress towards completing the project activities described in the work plan in a timely manner. The assistance agreement will include terms/conditions implementing this requirement.

L. Issuance of Awards: EPA reserves the right to negotiate appropriate changes in project terms and amounts (*i.e.*, changes that do not affect the integrity of the competition or materially change the application) consistent with EPA Order 5700.5A1 and other applicable policies, before making final decisions and awards. EPA reserves the right to reject all applications and make no awards. Applicants may be asked to include greater detail and specificity for their work plans before final awards are issued. Applicants may also be requested to satisfy data quality or peer review requirements before or shortly after the awarding of grants.

M. Data Access and Information Release. EPA has the right to obtain, reproduce, publish, or otherwise use the data first produced under the awards to be made under this solicitation and authorize others to receive, reproduce, publish, or otherwise use such data for Federal purposes under 40 C.F.R. Section 30.36(c). In addition, pursuant to 40 C.F.R. Section 30.36(d), if EPA receives a Freedom of Information Act request for research data that (1) relates to published research findings produced under an EPA award and (2) was used by the federal government in developing an agency action that has the force and effect of law, then EPA shall request, and the award recipient shall provide, within a reasonable time, the research data so that it may be made available to the public through procedures established under the FOIA.

N. Unpaid Federal Tax Liabilities and Felony Convictions For Non-Profit and For -Profit Organizations. Awards made under this announcement are subject to the provisions regarding unpaid federal tax liabilities and federal felony convictions contained in the Department of Interior, Environment, and Related Agencies Appropriation Act, 2012, HR 2055, Division E, Sections 433 and 434. These provisions prohibit EPA from awarding funds made available by the Act to any for-profit or nonprofit organizations: (1) subject to any unpaid federal tax liability that has been assessed, for which all judicial and administrative remedies have been exhausted or have lapsed, and that is not being paid in a timely manner pursuant to any agreement with the authority responsible for collecting the tax liability; or (2) that was convicted (or had an officer or agency of such corporation acting on its behalf convicted) of a felony criminal convictions under any federal law within 24 months preceding the award, unless EPA has considered suspension or debarment of the corporations, or such officer or agency, based on these tax liabilities or convictions, and determined that such action is not necessary to protect the government's interests. Organizations that are covered by these prohibitions are ineligible to receive an award under this announcement.

O. Website References in RFA. Any non-federal websites or website links included in this RFA are provided for proposal preparation and/or informational purposes only. EPA does not endorse any of these entities or their services. In addition, EPA does not guarantee that any linked, external websites referenced in this solicitation comply with Section 508 (Accessibility Requirements) of the Rehabilitation Act.

VII. AGENCY CONTACTS

General RFA Contact: (For administrative, eligibility, and other general RFA questions):

Patricia Thompson, Chief / 312-886-6015
Great Lakes National Program Office
Policy Coordination and Communications Branch

Michael Russ, Senior Program Advisor / 312-886-4013 /RFA2012@glnpo.net

Technical Contacts:

- Toxic Substances: Ted Smith / 312-353-6571 / smith.edwin@epa.gov

- Invasive Species: James Schardt 312-353-5085 / schardt.james@epa.gov

- Nearshore Health and Nonpoint Source Pollution: Elizabeth Hinchey-Malloy 312/886-3451 / Hinchey.elizabeth@epa.gov

- Accountability, Education, Monitoring, Evaluation, Communication, and Partnerships: Todd Nettesheim 312-353-9153 / nettesheim.todd@epa.gov

VIII. OTHER INFORMATION

Funding for state and tribal capacity for LaMPs and RAPs is being negotiated separately with states and Indian tribes. Organizations interested in funding for remediation of contaminated sediments should contact tuchman.marc@epa.gov.

GLNPO will send an e-mail announcement of these and any of its funding opportunities to all who register at http://www.epa.gov/greatlakes/maillist.

Appendix I
Lakewide Management Plan (LaMP) Priorities and Goals

The Great Lakes Water Quality Agreement (GLWQA) calls for the development of action-oriented strategic plans entitled Lakewide Management Plans (LaMPs), agreed to by binational Great Lakes resource managers to protect, restore and maintain the Great Lakes ecosystem. The LaMPs assess, restore, protect, and monitor the ecosystem health of each of the Great Lakes and are used to coordinate the work of all the government, tribal, and non-government partners working to improve the Lake ecosystems. A public consultation process is used to ensure that the LaMPs address the public's concerns. LaMPs for Lakes Ontario, Michigan, Erie, and Superior, the Lake Huron Binational Partnership Action Plan, and the Comprehensive Management Plan for Lake St. Clair identify the goals and priorities for these respective areas and are each considered LaMPs for the purposes of this RFA.

This Appendix summarizes the priorities and goals of the Lakewide Management Plans for Lakes Ontario, Michigan, Erie, and Superior, and the Lake Huron Binational Partnership Action Plan, as follows:

- Lake Superior Lakewide Management Plan goals, objectives and challenges can be viewed online at http://www.epa.gov/greatlakes/superior.html. Priorities include reduction of the nine critical pollutants in the Zero Discharge Demonstration Program; reductions of substances of emerging concern; implementation of the Lake Superior Ecosystem Goals and Objectives; implementation of the Lake Superior Aquatics Invasive Species Complete Prevention Plan; restoration and rehabilitation of native fish populations; habitat restoration and protection; watershed management and plan implementation; reducing mining impacts on the ecosystem; land use change; protection of biodiversity and climate change adaptation and mitigation efforts.

- Lake Huron Binational Partnership Action Plan priorities can be found online in the Lake Huron Binational Partnership Action Plan at http://www.epa.gov/greatlakes/lamp/lh_2008. Specific Lake Huron Binational Partnership priorities include a reduction of contaminants causing fish consumption advisories; efforts to improve beach quality and management of nearshore algae growth; an increased understanding of how nutrients and energy are partitioned and flow between the nearshore and offshore aquatic environment; restoration of the Lake Huron fishery, including lake herring as part of a forage base and lake sturgeon as a benthic feeder; restoring connectivity of tributaries while taking into account the possible movement of invasive species and contaminants; protection of cold water tributaries in recognition of a warming climate; and collaborative projects that encourage sustainable land-usage practices that improve the water quality and habitat of the Lake Huron ecosystem, including implementation of recommendations found in *The Sweet Water Sea - An International Biodiversity Conservation Strategy for Lake Huron,* (http://conserveonline.org/workspaces/lakehuron.bcs/documents/all.html).

- The Lake Michigan Lakewide Management Plan goals and sustainability targets, challenges and next steps can be viewed online at http://www.epa.gov/greatlakes/michigan.html. The GLRI topics and corresponding LaMP Chapters are: Toxic Substances (contaminated

sediments and fish effects): LaMP Chapters 1 and 7; Invasive Species (aquatic and terrestrial invasives): LaMP Chapter 8: Nearshore Health and Nonpoint Source Pollution (nutrient loadings and land use): LaMP and watershed fact sheets Chapters 2, 3, 6, 7, and 12; Habitat, Biodiversity and Wildlife Protection and Restoration (aquatic food web, wetlands, dunes and migration corridors): LaMP Chapter 4; and Accountability, Education, Monitoring, Evaluation, Communication and Partnerships: LaMP Chapters 9, 10, and 11. Current Lake Michigan LaMP priorities are near shore projects, invasive species, watershed management, and protecting biodiversity.

- The Lake Erie LaMP can be viewed online at http://www.epa.gov/greatlakes/lamp/le_2008. Specific Lake Erie LaMP priorities include the implementation of an increased number of actions and activities to reduce the loadings of nutrients (especially total phosphorus and/or soluble reactive phosphorus) to the lake; the development and implementation of watershed management actions to reduce phosphorus loadings to the Western Basin, and monitoring of those actions to determine their success; the development of actions that reduce or lead to the reduction of harmful algal blooms in the Western Basin of Lake Erie; the development of habitat strategies and actions that promote sustainable biodiversity throughout the Lake Erie ecosystem; projects and activities that increase public understanding of the importance of watershed management and how land links to the tributaries to the nearshore of the lake; and actions to encourage sustainable land-usage practices that improve the water quality and habitat of the Lake Erie ecosystem on a lake basin or watershed level.

- The Lake Ontario LaMP's updated chapters, including the workplan in Chapter 12, can be viewed online in "LaMP 2008" at: http://epa.gov/greatlakes/lamp/lo_2008/index.html. Also available at this site are the annual Lake Ontario Update activities reports. Additional chapters of the LaMP which were not updated in 2008 can be viewed in the "2006 Biennial Report" at http://epa.gov/greatlakes/lakeont/2006/index.html. Lake Ontario priorities include reduction of sources and loads of critical pollutants including emerging chemicals; contaminant track-down efforts; coordination of binational monitoring efforts, particularly those related to LaMP ecosystem indicators; restoration of bald eagle and native fish species; development and implementation of watershed management plans addressing nonpoint sources and other lake-wide issues; improved tributary monitoring in AOC and non-AOC areas including but not limited to, measurement of total phosphorus, soluble reactive phosphorus, total nutrients, chlorophyll a and clarity; development and implementation of a monitoring plan for the nearshore zone of Lake Ontario; development of a management plan to integrate AOC, nearshore and LaMP programs and projects; use of satellite imagery to track large-scale ecosystem changes such as thermal, suspended sediment, chlorophyll a, or other criteria; and implementation projects supporting the "Lake Ontario Binational Biodiversity Conservation Strategy Implementation Plan," which can be found online at: (http://www.epa.gov/greatlakes/lakeont/lake-ontario-biodiversity-conservation-strategy-201104.pdf).

- *The St Clair River and Lake St. Clair Comprehensive Management Plan* (Management Plan) is available on-line at http://www.glc.org/stclair. The Management Plan identifies goals and priorities for restoration and protection of Lake St. Clair. Lake St. Clair priorities include: 1) invasive species control; 2) habitat protection/restoration; 3) prevention/elimination of harmful discharges; and 4) development of information management that integrates modeling

with monitoring and provides real-time data. Recently, the Lake St. Clair/St. Clair River Partnership completed a Strategic Implementation Plan (SIP) that provides a five-year strategy for specific actions that implement Management Plan priorities and achieve Lake St. Clair goals. The SIP and more on actions to restore and protect Lake St. Clair can be found at http://www.semcog.org/lakestclair.

Appendix II
Grants.gov Submission Instructions

The electronic submission of your application through Grants.gov must be made by an authorized official representative (AOR) of your institution who is registered with Grants.gov. For more information, go to http://www.grants.gov and click on "Get Registered" on the left side of the page. *Note that the registration process may take a week or longer to complete.* If your organization is not currently registered with Grants.gov, we encourage them to designate an AOR and ask that individual to begin the registration process as soon as possible.

To begin the application process under this grant announcement, go to http://www.grants.gov and click on the "Apply for Grants" tab on the left side of the page. Then click on "Apply Step 1: Download a Grant Application Package" to download the compatible Adobe viewer and obtain the application package. **To apply through Grants.gov you must use Adobe Reader applications and download the compatible Adobe Reader version (Adobe Reader applications are available to download for free on the Grants.gov website. For more information on Adobe Reader please visit the Help section on Grants.gov at** http://www.grants.gov/help/help.jsp or http://www.grants.gov/aboutgrants/program_status.jsp). Once you have downloaded the viewer, you may retrieve the application package by entering the Funding Opportunity Number, EPA-R5-GL2012-1, or the CFDA number that applies to the announcement (CFDA 66.469), in the appropriate field. You may also be able to access the application package by clicking on the Application button at the top right of the synopsis page for this announcement on http://www.grants.gov (to find the synopsis page, go to http://www.grants.gov and click on the "Find Grant Opportunities" button on the left side of the page and then go to Search Opportunities and use the Browse by Agency feature to find EPA opportunities).

Application Submission Deadline: Your organization's AOR must submit your complete application electronically to EPA through Grants.gov (http://www.grants.gov) no later than 11:59 p.m. Eastern Daylight Time on May 24, 2012.

Please submit the application materials described below.

Application Materials:

1. Application for Federal Assistance (SF-424)
2. Budget Information for Non-Construction Programs (SF-424A)
3. Assurances for Non-Construction Programs (SF-424B)
4. Grants.gov Lobbying Form (6600-06)
5. EPA Key Contacts Form (5700-54)
6. EPA Form 4700-4 – Pre-award Compliance Review Report
7. Narrative Proposal-Project Narrative Attachment Form
8. Other Attachments Form - Resumes or *curriculum vitae* of Principal Investigators and Critical Staff
9. Disclosure of Lobbying Activities (SF-LLL)
10. Other Attachments Form - Negotiated Indirect Cost Rate Agreement

11. Other Attachments Form - Letters of support
12. Other Attachments Form - Meetings/Conferences/Workshops
13. Other Attachments Form - Overarching plan for which there is no Internet link (URL)
14. Other Attachments Form - Scientific peer review
15. Other Attachments Form - Maps or charts.

Application Preparation and Submission Instructions

The application package *must* include the following items:.
1. Standard Form (SF) 424, Application for Federal Assistance. Complete the form. There are no attachments. Please be sure to include the organization fax number and email address in Block 5 of the Standard Form SF 424. Please note that the organizational Dun and Bradstreet (D&B) Data Universal Number System (DUNS) number must be included on the SF-424. Organizations may obtain a DUNS number at no cost by calling the toll-free DUNS number request line at 1-866-705-5711.

2. SF-424A, Budget Information for Non-Construction Programs. Complete the form. There are no attachments. The total amount of federal funding requested for the project period should be shown on line 5(e) and on line 6(k) of SF-424A. If indirect costs are included, the amount of indirect costs should be entered on line 6(j). The indirect cost rate (i.e., a percentage), the base (e.g., personnel costs and fringe benefits), and the amount should also be indicated on line 22. If indirect costs are requested, a copy of the Negotiated Indirect Cost Rate Agreement must be submitted as part of the application package. (See instructions for document 10 below.)

3. SF-424B, Assurances for Non-Construction Programs. Complete the form. There are no attachments.

4. Grants.gov Lobbying Form – Certification Regarding Lobbying (6600-06). Complete the form. There are no attachments.

5. EPA Form 5700-54, Key Contacts Form. Complete the form. There are no attachments. If additional pages are needed, attach these additional pages to the electronic application package by using the “Other Attachments Form” in the “Optional Documents” box. (See Application Preparation and Submission Instructions below for more details.)

6. EPA Form 4700-4, Pre-Award Compliance Review Report. Complete the form.

7. Narrative Proposal. Includes the Summary Information Page, Workplan, and Detailed Budget Narrative. Prepare as described in **Section IV.C.2** of the announcement.

8. Other Attachments Form - Resumes or curriculum vitae of Principal Investigators and critical staff. Use the “Other Attachments Form” in the “Optional Documents” box to attach a copy of the resume or curriculum vitae of principal investigators and critical staff for the proposed project. Such documentation should outline the education, work history, and knowledge/expertise of the individual that relate to managing the proposed project.

9. SF-LLL, Disclosure of Lobbying Activities, if applicable. This form is required if your organization is involved in lobbying. Complete the form if your organization is involved in lobbying activities.

10. Other Attachments Form – Negotiated Indirect Cost Rate Agreement. Use if indirect costs are included in the project budget. Use the “Other Attachments Form” in the “Optional Documents” box to

attach a copy of your organization's Indirect Cost Rate Agreement, if applicable. (See Application Preparation and Submission Instructions below for more details.) You must submit a copy of your organization's Indirect Cost Rate Agreement as part of the application package if your proposed budget includes indirect costs.

11. Other Attachments Form – Support Letters. Use the "Other Attachments Form" in the "Optional Documents" box to attach any relevant letters from collaborators or other letters of support. A letter of support may also be required for voluntary cost share. Specifically indicate how the supporting organization will assist in the project or what that organization supports, as applicable.

12. Other Attachments Form - Meetings/Conferences/Workshops. If you plan to host meetings, conferences or workshops please include information such as: Who is initiating the meeting? How will it be advertised? Whose logo will be on the agenda and materials? What is the percentage distribution of the persons attending (*i.e*. percent federal government, tribal members, public participants, state, local)? Will you be preparing the proceedings or analysis and disseminate this information back to the appropriate community? Do you anticipate any program income being generated, including registration fees?

13. Other Attachments Form – Overarching Plan. Use the "Other Attachments Form" in the "Optional Documents" box to attach a copy of any applicable Overarching Plan for which there is no Internet link (URL). See RFA Section IV.2.b.iii for additional information.

14. Other Attachments Form – Scientific Peer Review. Use the "Other Attachments Form" in the "Optional Documents" box to attach any applicable scientific peer review of the project that has already been completed.

15. Other Attachments Form – Maps or Charts. Use the "Other Attachments Form" in the "Optional Documents" box to attach any applicable maps or charts.

Documents 1 through 7 listed under Application Materials above should appear in the "Mandatory Documents" box on the Grants.gov Grant Application Package page.
For documents 1 through 6, click on the appropriate form and then click "Open Form" below the box. The fields that must be completed will be highlighted in yellow. Optional fields and completed fields will be displayed in white. If you enter an invalid response or incomplete information in a field, you will receive an error message. When you have finished filling out each form, click "Save." When you return to the electronic Grant Application Package page, click on the form you just completed, and then click on the box that says, "Move Form to Submission List." This action will move the document over to the box that says, "Mandatory Completed Documents for Submission."

For document 7, you will need to attach electronic files. Prepare your narrative proposal as described above and in Section IV.C. and save the documents to your computer as a PDF file. When you are ready to attach your proposal to the application package, click on "Project Narrative Attachment Form," and open the form. Click "Add Mandatory Project Narrative File," and then attach your proposal (previously saved to your computer) using the browse window that appears. You may then click "View Mandatory Project Narrative File" to view it. Enter a brief descriptive title of your project in the space beside "Mandatory Project Narrative File Filename;" the filename should be no more than 40 characters long. If there are other attachments to submit to accompany your proposal, you may click "Add Optional Project Narrative File" and proceed as before. When you have finished attaching the necessary documents, click "Close Form." When you return to the "Grant Application Package" page, select the "Project Narrative Attachment Form" and click "Move Form to Submission List." The form should now appear in the box that says, "Mandatory Completed Documents for Submission."

Documents 8 through 15 are submitted under the "Optional Documents" box, but ***please note that these so-called "optional" documents must also be submitted as part of the application package, if applicable to your organization or the category to which you are submitting your application.*** You must submit document 8 – Resumes or curriculum vitae of Principal Investigators and critical staff. You are only required to submit document 9 – SF-LLL, Disclosure of Lobbying Activities – if your organization is involved in lobbying activities. You are required to submit document 10 – Negotiated Indirect Cost Rate Agreement – if you have included any indirect costs in your proposed budget. You are required to submit document 11 – Support Letters – if necessary to confirm non-applicant cost share; however it may also be submitted demonstrating evidence of collaboration and support. Items 13-15 are optional. To attach documents 8-15, use the "Other Attachments Form" in the "Optional Documents" box. After attaching the documents, please remember to highlight the "Other Attachments Form" and click "Move Form to Submission List" in order to move the documents to the box that says, "Optional Completed Documents for Submission."

Once you have finished filling out all of the forms/attachments and they appear in one of the "Completed Documents for Submission" boxes, click the "Save" button that appears at the top of the Web page. It is suggested that you save the document a second time, using a different name, since this will make it easier to submit an amended package later if necessary. Please use the following format when saving your file: "Applicant Name – FY12 – Focus Area.Category[10] – 1st Submission" or "Applicant Name – FY12 – Focus Area.Category – Back-up Submission." If it becomes necessary to submit an amended package at a later date, then the name of the 2nd submission should be changed to "Applicant Name – FY12 – Focus Area.Category– 2nd Submission."

Once your application package has been completed and saved, send it to your AOR for submission to EPA through Grants.gov. Please advise your AOR to close all other software programs before attempting to submit the application package through Grants.gov.

In the "Application Filing Name" box, your AOR should enter your organization's name (abbreviate where possible), the fiscal year (*e.g.*, FY12), and the grant category (*e.g.*, TX.P2, IS.Prevent, IS.Control, IS.Detect, NS.Beach, NS.Remed, NS.Tech, AEMECP.CC, or AEMECP.LaMP). The filing name should

[10] In your submission, replace "Focus Area.Category" with the applicable abbreviation below to the right:

	Category in RFA	Focus Area.Category
**I.A. Toxic Substances**		
	I.A.1. Toxic Substance Reduction	TX.Reduction
**I.B. Invasive Species**		
	I.B.1. Invasive Species Prevention	IS.Prevent
	I.B.2. Early Detection Technology for High Risk Invasive Species	IS.Control
	I.B.3 Invasive Species Control	IS.Detect
**I.C. Nearshore Health and Nonpoint Source Pollution**		
	I.C.1. Making Beaches Safer	NS.Beach
	I.C.2. Watershed Remediation	NS.Remed
	I.C.3 Increasing Technical Expertise to Accelerate Nutrient Reduction	NS.Tech
**I.D. Accountability, Education, Monitoring, Evaluation, Communication, and Partnerships**		
	I.D.1. Increasing Climate Change Resiliency in Great Lakes Communities	AEMECP.CC
	I.D.2. Implementing Lakewide Management Plan Programs and Projects	AEMECP.LaMP

not exceed 40 characters. From the "Grant Application Package" page, your AOR may submit the application package by clicking the "Submit" button that appears at the top of the page. The AOR will then be asked to verify the agency and funding opportunity number for which the application package is being submitted. If problems are encountered during the submission process, the AOR should reboot his/her computer before trying to submit the application package again. [It may be necessary to turn off the computer (not just restart it) before attempting to submit the package again.] If the AOR continues to experience submission problems, he/she may contact Grants.gov for assistance by phone at 1-800-518-4726 or email at http://www.grants.gov/help/help.jsp or he/she may contact Michael Russ via email at RFA2012@glnpo.net

Applications submitted through http://www.grants.gov will be time and date stamped electronically.

Appendix III
Budget Sample

Budget Detail

This section of the work plan is a detailed description of the budget found in the SF-424A, and must include a detailed discussion of how EPA funds will be used. Applicants must **itemize** costs related to personnel, fringe benefits, travel, equipment, supplies, contractual costs, other direct costs, indirect costs, and total costs.

If the project budget includes any voluntary cost share, the Budget Detail portion of the narrative proposal must include a detailed description of how the applicant will obtain the cost-share and how the cost-share funding will be used. If EPA accepts an offer for a voluntary cost-share, applicants must meet their sharing commitment as a legal condition of receiving EPA funding. If the proposed cost-share is to be provided by a third-party, a letter of commitment is required. Any form of cost-share included in the Budget Detail must also be included on the SF 424 and SF 424A. Please see Sections III and Section IV.C.2.B.iii of this RFA for more detailed information on cost-share.

Applicants should use the following instructions, budget object class descriptions, and example table to complete the Budget Detail section of the work plan. Use only whole dollar amounts.

☐**Personnel - List all staff positions by title. Give annual salary, percentage of time assigned to the project, and total cost for the budget period.** This category includes only direct costs for the salaries of those individuals who will perform work directly for the project (generally, paid employees of the applicant organization). If the applicant organization is including staff time (in-kind services) as a cost share, this should be included as Personnel costs. Personnel costs do not include: (1) costs for services of consultants, contractors, consortia members, or other partner organizations, which are included in the "Contractual" category; (2) costs for employees of subrecipients under subawards, which are included in the "Other" category; or (3) effort that is nor directly in support of the proposed project, which may be covered by the organization's negotiated indirect cost rate. The budget detail must identify the personnel category type by Full Time Equivalent (FTE), including percentage of FTE for part-time employees, number of personnel proposed for each category, and the estimated funding amounts.

☐**Fringe Benefits - Identify the percentage used, the basis for its computation, and the types of benefits included**. Fringe benefits are allowances and services provided by employers to their employees as compensation in addition to regular salaries and wages. Fringe benefits include, but are not limited to the cost of leave, employee insurance, pensions and unemployment benefit plans.

☐**Travel - Specify the mileage, per diem, estimated number of trips in-State and out-of-State and international (include specific international locations), number of travelers, and other costs for each type of travel.** Travel may be integral to the purpose of the proposed project (e.g. inspections) or related to proposed project activities (*e.g.*, attendance at meetings).

Travel costs do not include: (1) costs for travel of consultants, contractors, consortia members, or other partner organizations, which are included in the "Contractual" category; (2) travel costs for employees of subrecipients under subawards, which are included in the "Other" category.

☐**Equipment - Identify each item to be purchased which has an estimated acquisition cost of $5,000 or more per unit and a useful life of more than one year.** Equipment also includes accessories necessary to make the equipment operational. Equipment does not include: (1) equipment planned to be leased/rented, including lease/purchase agreement; or (2) equipment service or maintenance contracts. These types of proposed costs should be included in the "Other" category. Items with a unit cost of less than $5,000 should be categorized as supplies, pursuant to 40 CFR Sections 31.3 and 30.2. The budget detail must include an itemized listing of all equipment proposed under the project.

☐**Supplies - "Supplies" means all tangible personal property other than "equipment".** The budget detail should identify categories of supplies to be procured (e.g., laboratory supplies or office supplies). Non-tangible goods and services associated with supplies, such as printing service, photocopy services, and rental costs should be included in the "Other" category.

☐**Contractual - Identify each proposed contract and specify its purpose and estimated cost.** Contractual/consultant services are those services to be carried out by an individual or organization, other than the applicant, in the form of a procurement relationship. Leased or rented goods (equipment or supplies) should be included in the "Other" category. The applicant should list the proposed contract activities along with a brief description of the scope of work or services to be provided, proposed duration, and proposed procurement method (competitive or noncompetitive), if known.

☐**Other - List each item in sufficient detail for EPA to determine the reasonableness and allowability of its cost.** This category should include only those types of direct costs that do not fit in any of the other budget categories. Examples of costs that may be in this category are: insurance, rental/lease of equipment or supplies, equipment service or maintenance contracts, printing or photocopying, rebates, and subaward costs. Subawards (*e.g.*, subgrants) are a distinct type of cost under this category. The term "subaward" means an award of financial assistance (money or property) by any legal agreement made by the recipient to an eligible subrecipient. This term does not include procurement purchases, technical assistance in the form of services instead of money, or other assistance in the form of revenue sharing, loans, loan guarantees, interest subsidies, insurance, or direct appropriations. Subcontracts are not subawards and belong in the contractual category. Applicants must provide the aggregate amount they propose to issue as subaward work and a description of the types of activities to be supported.

☐**Indirect Charges - If indirect charges are budgeted, indicate the approved rate and base.** Indirect costs are those incurred by the grantee for a common or joint purpose that benefit more than one cost objective or project, and are not readily assignable to specific cost objectives or projects as a direct cost. In order for indirect costs to be allowable, the applicant must have a federal or state negotiated indirect cost rate (*e.g.*, fixed, predetermined, final or provisional), or must have submitted a proposal to the cognizant federal or state agency. Examples of Indirect Cost Rate calculations are shown below:

o Personnel (Indirect Rate x Personnel = Indirect Costs)

o Personnel and Fringe (Indirect Rate x Personnel & Fringe = Indirect Costs)
o Total Direct Costs (Indirect Rate x Total direct costs = Indirect Costs)
o Direct Costs minus distorting or other factors such as contracts and equipment (Indirect Rate x (total direct cost – distorting factors) = Indirect Costs)

Example Budget Table

	EPA Funding	Cost-Share
**Personnel**		
(1) Project Manager @ $40/hr x 10 hrs/week x 52 wks		$20,800
(5) Project Staff @ $30/hr x 40 hrs/week x 40 wks	$244,000	
TOTAL PERSONNEL	$244,000	$20,800
**Fringe Benefits**		
20% of Salary and Wages	20%($244,000)	20%(20,800)
- Retirement, Health Benefits, FICA, SUI	$48,800	$4,160
TOTAL FRINGE BENEFITS	$48,800	$4,160
**Travel**		
In State travel for Project Manager and staff: 500 mi/mo @ $0.55/mi x 12 mos.	$3,300	
Out of State (IL, WI, IA)Travel for Project Staff: 20 trips per month x $2,500 per trip	$600,000	
SOLEC Meeting (Toronto, Canada) Travel for Project Manager: 2 trips/year x $3,500 each	$7,000	
TOTAL TRAVEL	$610,300	
**Equipment**		
Transducer, coupling, and software package	$25,700	
Electrofishing boom shocker (2 x $7,500each)	$15,000	
1 Project Vehicle	$25,000	
1 Project Boat	$15,000	
TOTAL EQUIPMENT	81,100	
**Supplies**		
Office and related supplies to support training	$400	
Office computer and printer	$2,500	
TOTAL SUPPLIES	$2,900	
**Contractual**		
ABC Support Services Contract	$100,000	
XYZ Land & Water Conservation	$66,400	
TOTAL CONTRACTUAL	$166,400	
**Other**		
Travel for 3 representatives to attend workshop training – 100 trips x $1,000 each	$100,000	
Travel for 4 representatives to attend workshop training – 200 trips x $2,000 each	$400,000	
TOTAL OTHER	$500,000	
**Indirect Charges**		
Federal Negotiated Indirect Cost Rate = 10% (Indirect Rate x Personnel = Indirect Costs; as negotiated)	$26,480	
TOTAL INDIRECT	$26,480	
TOTAL FUNDING	$1,679,580	$24,960
TOTAL PROJECT COST	$1, 704,540	

** Any voluntary cost-share funds, while not required under this RFA, must also be included on the SF-424A as detailed in Section IV.C.2.B.iii of this RFA. Federal funds are not allowed to be used for cost share; please identify the source of the cost share in your budget narrative.

Note on Management Fees: When formulating budgets for proposals, applicants must not include management fees or similar charges in excess of the direct costs and indirect costs at the

rate approved by the applicant’s cognizant federal audit agency, or at the rate provided for by the terms of the agreement negotiated with EPA. The term "management fees or similar charges" refers to expenses added to the direct costs in order to accumulate and reserve funds for ongoing business expenses, unforeseen liabilities, or for other similar costs that are not allowable under EPA assistance agreements. Management fees or similar charges cannot be used to improve or expand the project funded under this agreement, except to the extent authorized as a direct cost of carrying out the work plan**.**

Expeditious Spending and Sufficient Progress in the use of GLRI Funds: Include an explanation of how, if the applicant is awarded a grant, they will ensure that the funding will be used expeditiously.
