# Supplementary material for: Understanding Acceptable Level of Risk: Incorporating the Economic Cost of Under-Managing Invasive Species
Source: PLoS One. 2015 Nov 4;10(11):e0141958. doi: 10.1371/journal.pone.0141958 (PMC4633185; doi:10.1371/journal.pone.0141958)
Supplement: S2 Supplementary Information — (DOCX) [file pone.0141958.s003.docx]

S2 Supplementary Information

Data related to: “For the current budget scenario it was based on the MI DEQ monitoring budget for macrophytes (US$64,137/annum)”:

This was a personal communication with the DEQ Aquatic Invasive Species Department.
