# Supplementary material for: Understanding Acceptable Level of Risk: Incorporating the Economic Cost of Under-Managing Invasive Species
Source: PLoS One. 2015 Nov 4;10(11):e0141958. doi: 10.1371/journal.pone.0141958 (PMC4633185; doi:10.1371/journal.pone.0141958)
Supplement: S3 Supplementary Information — (PDF) [file pone.0141958.s004.pdf]

All sales, single family homes, state of Michigan, sold via SWMRIC system, lake frontage, March 1-June 9, 2014
Calculations at Bottom.
© Pete Bruinsma Realtor 2014, www.PeteBruinsma.com

Street Full Address	Selling Price
2905 Bonnell Ave SE East Grand Rapids, MI 49506	$7,800,000.00
18777 N Fruitport Rd Spring Lake, MI 49456	$2,600,000.00
2234 Lakeshore Dr Long Beach, IN 46360	$2,325,000.00
872 Monroe Blvd South Haven, MI 49090-1622	$1,925,000.00
20683 Decatur Dr Cassopolis, MI 49031	$1,730,000.00
520 Marquette Dr New Buffalo, MI 49117	$1,500,000.00
1584 Waukazoo Dr Holland, MI 49424	$1,175,000.00
1726 N Darling Ln Glenn, MI 49416	$1,100,000.00
648 Manhattan Rd SE East Grand Rapids, MI 49506	$1,100,000.00
19085 North Shore Dr Spring Lake, MI 49456	$1,050,000.00
19663 North Shore Dr Spring Lake, MI 49456	$969,000.00
16974 Landing Dr Spring Lake, MI 49456	$930,000.00
8 Circle Dr Fruitport, MI 49415	$900,000.00
8484 Heron Vw NE Rockford, MI 49341	$865,000.00
6076 W Bay Ct Hudsonville, MI 49426	$850,000.00
8382 Windermere Watervliet, MI 49098	$835,000.00
4095 Reeds Lake Blvd SE Grand Rapids, MI 49506	$825,000.00
76568 Fieldstone Cir South Haven, MI 49090	$800,000.00
895 South Shore Dr Holland, MI 49423	$787,500.00
107 Crestwood Dr Holland, MI 49424	$760,000.00
17302 Beach Ridge Way West Olive, MI 49460	$755,500.00
18201 N Fruitport Rd Spring Lake, MI 49456	$749,900.00
68656 West Banks Dr Edwardsburg, MI 49112	$749,000.00
62117 Lake St Cassopolis, MI 49031	$745,000.00
5400 Waldorf Rd Delton, MI 49046	$740,000.00
5015 S Anderfind Dr Ludington, MI 49431	$725,952.00
492 E Gull Lake Dr Augusta, MI 49012	$710,000.00
839 S Lakeshore Dr Ludington, MI 49431	$703,000.00
10655 Wildwood Ln Richland, MI 49083	$700,000.00
46444 Meadow Ln Lawrence, MI 49064	$675,000.00
528 Chester St Pentwater, MI 49449	$653,125.00
3883 Forest Trl Allegan, MI 49010	$650,000.00
7745 Margaret West Olive, MI 49460	$645,000.00
12508 Park Dr Wayland, MI 49348	$629,000.00
566 Howard Ave Holland, MI 49424	$615,000.00
6915 Old Channel Trl Montague, MI 49437	$610,000.00
1835 Van Buren St Hudsonville, MI 49426	$591,000.00
835 Tostenabe Ln North Muskegon, MI 49445	$590,000.00
6312 Westlake Rd Bellevue, MI 49021	$555,000.00
63486 Birch Rd Vandalia, MI 49095	$545,000.00
113 Back Bay Pt Coldwater, MI 49036	$525,000.00
2619 Lake Shore Dr Niles, MI 49120	$525,000.00
530 Glenwood Ave Muskegon, MI 49445	$520,000.00
840 Blue Star Hwy South Haven, MI 49090	$520,000.00
17409 Lake Beach Dr Spring Lake, MI 49456	$515,000.00

2236 Van Singel Lake Dr SW Byron Center, MI 49315	$510,000.00
15700 Willows Dr Spring Lake, MI 49456-1145	$500,000.00
13455-A 7 Mile Rd NE Belding, MI 49341	$500,000.00
2200 Sunset Dr Fennville, MI 49408	$500,000.00
263 Norwood Ave Holland, MI 49424	$490,000.00
103 Crestwood Dr Holland, MI 49424	$489,000.00
2696 Lakeshore Dr Hillsdale, MI 49242	$485,000.00
4128 Eagle Rock Ct SW Grandville, MI 49418	$476,000.00
1458 Burlington Hickory Corners, MI 49060	$465,000.00
31112 Curran Beach Rd Dowagiac, MI 49047	$465,000.00
730 Fall St Spring Lake, MI 49456	$465,000.00
3092 Lakeshore Ave Benton Harbor, MI 49022	$460,000.00
61797 Crestlane Dr Sturgis, MI 49091	$460,000.00
302 E Kott Rd Manistee, MI 49660	$455,000.00
2200 Van Singel Lake Byron Center, MI 49315	$450,000.00
69590 Shady Ln White Pigeon, MI 49099	$450,000.00
4160 Harbor Point Dr Muskegon, MI 49441	$450,000.00
3131 Elmwood Beach Middleville, MI 49333	$440,000.00
61526 Crystal Beach Rd Sturgis, MI 49091	$435,000.00
4335 N Ridge Rd Pentwater, MI 49449	$430,000.00
155 Lynwood Dr Battle Creek, MI 49015	$428,000.00
401 Harrington Rd Delton, MI 49046	$424,000.00
11319 Elizabeth Dr Three Rivers, MI 49093	$420,000.00
2120 Midlake Dr Hickory Corners, MI 49060	$420,000.00
1126 Lakeshore Dr Michigan City, IN 46360	$420,000.00
6143 Belding Rd Rockford, MI 49341	$412,000.00
71384 Indiana Lake Dr Union, MI 49130	$400,000.00
678 Lester Rd Coldwater, MI 49036	$395,000.00
18797 N Fruitport Rd Spring Lake, MI 49456	$395,000.00
5420 W Apple Ln Ludington, MI 49431	$389,000.00
62187 Lagoon Cassopolis, MI 49031	$385,000.00
739 Ruddiman Dr Muskegon, MI 49445	$385,000.00
15686 Connelly Ave Spring Lake, MI 49456	$382,900.00
355 Horseshoe Ct Plainwell, MI 49080	$382,500.00
7215 Buck Lake Woods Dr SE Alto, MI 49302	$379,000.00
8693 Cedar Lake Dr Jenison, MI 49428	$378,301.00
1410 Timber Trail Dr Whitehall, MI 49461	$375,000.00
9411 Fraulin Dr Richland, MI 49083	$375,000.00
14844 Elm Dr Marcellus, MI 49067	$375,000.00
3303 Branisha Dr NE Grand Rapids, MI 49525-7506	$375,000.00
2319 Lorraine Ave Kalamazoo, MI 49008	$375,000.00
12923 Canal View Dr Wayland, MI 49348	$372,450.00
62181 41st St Paw Paw, MI 49079	$370,000.00
70950 Lakeview Dr White Pigeon, MI 49099	$370,000.00
11172 Point Rd Three Rivers, MI 49093	$360,000.00
54963 Moody Dr Eau Claire, MI 49111	$345,000.00
6215 N Big Pine Dr Irons, MI 49644	$340,000.00
50926 3rd St Dowagiac, MI 49047	$337,500.00
58795 Edgewood Dr Three Rivers, MI 49093	$335,000.00
10335 Lincoln Lake Ave NE Greenville, MI 48838	$335,000.00
55548 N Fisher Lake Rd Three Rivers, MI 49093-9092	$335,000.00

8900 East Shore Dr Portage, MI 49002	$330,000.00
16299 Oak St Decatur, MI 49045	$330,000.00
4254 Winding Way Kalamazoo, MI 49004	$329,900.00
1961 Camp Madron Rd #9 Buchanan, MI 49107	$325,000.00
8925 W Pq Ave Mattawan, MI 49071	$325,000.00
733 Blackfoot Ct Coldwater, MI 49036	$324,000.00
702 Wayne Beach Ln Coldwater, MI 49036	$323,000.00
11355 Brielle Ln Nunica, MI 49448	$322,900.00
6867 Island Ct Coloma, MI 49038	$321,000.00
901 Treasure Island Dr Mattawan, MI 49071-9424	$320,000.00
2110 Ames Portage, MI 49002	$320,000.00
1451 Brookwood Dr Muskegon, MI 49441	$320,000.00
3200 Red Apple Rd Manistee, MI 49660	$317,500.00
1800 Antisdale Rd Muskegon, MI 49441	$313,000.00
1769 S Crooked Lake Dr Kalamazoo, MI 49009	$310,000.00
4523 Northwind Dr Delton, MI 49046	$305,000.00
7079 Davies Dr NE Rockford, MI 49341	$300,000.00
11333 E Royal Rd Canadian Lakes, MI 49346	$300,000.00
11484 Handy Ln Plainwell, MI 49080-9027	$300,000.00
23219 N Park St Edwardsburg, MI 49112-9575	$296,500.00
712 Lake Dr Muskegon, MI 49445	$295,000.00
6025 N Sherman Rd Ludington, MI 49431	$292,520.00
10860 Longpoint Dr Plainwell, MI 49080	$290,000.00
11297 Red Bud Trl Berrien Springs, MI 49103	$287,000.00
1685 Fairview Dr Allegan, MI 49010	$286,750.00
439 Lakeshore Muskegon, MI 49444	$285,000.00
17606 Simmons Ave NE Cedar Springs, MI 49319	$282,500.00
1411 Ruddiman Dr North Muskegon, MI 49445	$280,000.00
89 Riverview Dr Greenville, MI 48838	$280,000.00
446 Edgewater Ct Coldwater, MI 49036	$279,900.00
4631 Morning Glory Dr Reading, MI 49274	$277,000.00
11350 Long Point Dr Plainwell, MI 49080-9207	$274,900.00
11301 Crooked Lake NE Rockford, MI 49341	$271,800.00
6818 S Lakeshore Dr Pentwater, MI 49449	$270,000.00
7337 Pine Bay Dr NE Comstock Park, MI 49321	$270,000.00
9567 Sterling Richland, MI 49083	$270,000.00
6840 Kitson Rockford, MI 49341	$270,000.00
17923 112th Ave Nunica, MI 49448	$269,900.00
223 Lakeside Dr Quincy, MI 49082	$266,500.00
11087 Island Ct Allendale, MI 49401	$265,900.00
1220 Timber Trail Dr Whitehall, MI 49461	$265,000.00
3742 Ramshorn Dr Fremont, MI 49412	$259,900.00
13066 Blueberry Ln Holland, MI 49424	$259,000.00
183 S Lake Doster Dr Plainwell, MI 49080	$258,000.00
17614 Simmons Ave NE Cedar Springs, MI 49319	$258,000.00
2671 Crescent Beach Rd Manistee, MI 49660	$258,000.00
9258 Elmwood Ct #180 Canadian Lakes, MI 49346	$255,000.00
94610 CR 690 Dowagiac, MI 49047	$255,000.00
3844 Highgate Rd Muskegon, MI 49441	$255,000.00
7099 Oakshore Dr Twin Lake, MI 49457	$254,500.00
11554 W 179 Hwy Middleville, MI 49333	$254,000.00

7437 Decosta Dr NE Rockford, MI 49341	$250,000.00
5026 Gahan Ave NE Rockford, MI 49341	$250,000.00
57530 Cool Water Ln Colon, MI 49040	$250,000.00
61339 Timberlane Dr Jones, MI 49061	$247,000.00
10633 Wildwood Ln Richland, MI 49083-8534	$247,000.00
480 Hayes Ave Holland, MI 49424	$243,000.00
68397 George Smith Ct Edwardsburg, MI 49112	$242,000.00
11966 Mac Dr NE Belding, MI 48809	$240,000.00
10964 Crawford Lake Trl NE Cedar Springs, MI 49319	$240,000.00
568 Acorn Trl Horton, MI 49246	$240,000.00
360 Barber Dr Hillsdale, MI 49242	$238,000.00
1917 E Hess Lake Dr Newaygo, MI 49337	$237,500.00
68652 Blanchard St Sturgis, MI 49091	$237,000.00
2279 Florida Ln Camden, MI 49232	$236,500.00
10217 Van Hoose Rd Greenville, MI 48838	$235,500.00
5830 Jefferson Ave Muskegon, MI 49442	$235,000.00
7680 N Indian Lake Dr Scotts, MI 49088	$232,000.00
46502 Lakeview Dr Decatur, MI 49045	$230,000.00
333 Lakeshore Dr Battle Creek, MI 49015	$230,000.00
14322 Park Dr Mecosta, MI 49332	$230,000.00
14211 Percy Dr Mecosta, MI 49332	$226,000.00
67050 95th Ave Dowagiac, MI 49047	$225,000.00
68193 Christiana Dr Edwardsburg, MI 49112	$225,000.00
11732 5 Mile Rd NE Lowell, MI 49331	$225,000.00
27175 Baker Dr Sturgis, MI 49091-9152	$225,000.00
8860 Brule St #154 Canadian Lakes, MI 49346	$224,900.00
4787 Baker Rd Gobles, MI 49055	$220,000.00
719 Maple St Colon, MI 49040	$220,000.00
3656 118th Ave Allegan, MI 49010	$220,000.00
3737 Lake Shore Dr Shelbyville, MI 49344	$218,153.00
2239 Blue Goose Dr Fennville, MI 49408-9421	$218,000.00
2511 Country Club Way Albion, MI 49224	$217,000.00
665 Gilead Shores Dr Bronson, MI 49028	$215,000.00
337 S Gull Lake Dr Richland, MI 49083	$215,000.00
7895 S Crooked Lake Dr Delton, MI 49046	$214,000.00
2272 Ramblin Battle Creek, MI 49014	$214,000.00
8459 Pine Point Dr Newaygo, MI 49337	$213,000.00
239 Sunnyview St Grandville, MI 49418	$210,000.00
23664 Lakeshore Dr Gobles, MI 49055	$210,000.00
13291 Nielsen Dr Trufant, MI 49347	$208,000.00
516 Iyopawa Rd Coldwater, MI 49036	$207,500.00
15601 Lakeview Dr Buchanan, MI 49107	$205,500.00
52483 Joy Dr Dowagiac, MI 49047	$205,000.00
1243 Rose Dr Niles, MI 49120	$205,000.00
17020 White Rd Bailey, MI 49303	$202,000.00
11141 E Royal Rd Stanwood, MI 49346	$200,500.00
507 North St SE Caledonia, MI 49316	$200,000.00
2285 Elm St Fennville, MI 49408	$200,000.00
6698 Birch Shore Dr Lakeview, MI 48850	$200,000.00
32741 Lake Dr Lawton, MI 49065	$200,000.00
6886 W Winchester Dr Baldwin, MI 49304	$199,900.00

1724 N Lakeview Dr Mears, MI 49436	$199,000.00
3232 22 Mile Rd Sand Lake, MI 49343	$197,761.00
3576 N Karen St Hart, MI 49420	$197,000.00
32 W Duck Lake Dr Gobles, MI 49055	$195,000.00
11667 Olive Lake Dr West Olive, MI 49460	$194,000.00
8801 Waruf Ave Portage, MI 49002	$193,000.00
7667 W Royal Rd #632 Canadian Lakes, MI 49346	$187,000.00
31422 Hemlock Island St Dowagiac, MI 49047	$186,550.00
4336 Mayaka Ct SW Grandville, MI 49418	$185,250.00
11630 Sandy Bottom Rd NE Greenville, MI 48838	$181,000.00
2010 Island Dr Wayland, MI 49348	$180,000.00
1683 Edgewater St Muskegon, MI 49441	$180,000.00
46944 Meadow Ln Decatur, MI 49045	$179,000.00
354 Mud Lake Coldwater, MI 49036	$178,750.00
112 W North Lane Dr Newaygo, MI 49337	$178,500.00
3108 East Shore Dr Portage, MI 49002	$177,500.00
11600 Spring Point Dr Plainwell, MI 49080	$175,000.00
6081 W Storm Rd Ludington, MI 49431	$174,200.00
5121 S Long Lake Dr Portage, MI 49002	$172,000.00
10189 E Blue Lake Dr #33 Mecosta, MI 49332	$170,000.00
4478 S Sherman Ave Fremont, MI 49412	$170,000.00
17281-A E Suwanee Trl Howard City, MI 49329	$170,000.00
10931 Jordan Ct Allendale, MI 49401	$169,900.00
1931 Laraway Lake Dr SE Grand Rapids, MI 49546	$168,000.00
14711 E Horsehead Lake Dr Mecosta, MI 49332	$168,000.00
10971 Sixth St #35 Canadian Lakes, MI 49346	$168,000.00
94 Plum Hart, MI 49420	$167,500.00
6100 Oakridge Jonesville, MI 49250	$167,500.00
13111 E Englewright Dr Sand Lake, MI 49343	$167,000.00
713 Wigwam Way White Cloud, MI 49349	$165,000.00
4056 Causeway Dr Lowell, MI 49331	$165,000.00
11767 Fair Lake Dr Delton, MI 49046	$165,000.00
48101 CR 380 Grand Junction, MI 49056	$165,000.00
69259 Sunset Blvd Union, MI 49130	$165,000.00
12000 Southgate Dr Plainwell, MI 49080	$163,900.00
5000 Gordon Ave Newaygo, MI 49337	$160,000.00
328 W Hickory Battle Creek, MI 49017	$159,100.00
13667 West Point Dr Gowen, MI 49326	$158,000.00
192 Archer Vw Quincy, MI 49082	$158,000.00
14487 W Diane Dr Camden, MI 49232-9551	$158,000.00
330 Lake St Berrien Springs, MI 49103	$155,000.00
2811 Hilltop Kalamazoo, MI 49048	$155,000.00
15065 Becker Dr Mecosta, MI 49332	$155,000.00
69193 Center St Paw Paw, MI 49079	$155,000.00
7636 W Birch Ln New Era, MI 49446	$153,000.00
271 Rainbow Lake Dr Trufant, MI 49347	$153,000.00
4046 Lake Dr Allegan, MI 49010	$151,000.00
151 Dons Dr Quincy, MI 49082	$150,000.00
4719 S Sylvan Blvd Newaygo, MI 49337	$150,000.00
1229 Hess Lake Dr Grant, MI 49327	$149,900.00
6300 Springbrook Rd Horton, MI 49246	$149,000.00

2166 Parker Dr Wayland, MI 49348	$147,700.00
902 W Shore Rd Bronson, MI 49028	$147,000.00
351 Lake Dr Six Lakes, MI 48886	$145,900.00
6352 Oakridge Dr Jonesville, MI 49250	$145,000.00
705 E Hess Lake Dr Grant, MI 49327	$145,000.00
10284 Tomkinson Dr Scotts, MI 49088	$145,000.00
511 Tompkins Dr Coldwater, MI 49036	$145,000.00
11579 Crystal Lake Jerome, MI 49249	$144,000.00
11338 Harmon Three Rivers, MI 49093	$143,000.00
12205 Bair Lake St Jones, MI 49061	$142,500.00
6929 Wood Rd Muskegon, MI 49444	$138,000.00
11246 Long Point Dr Plainwell, MI 49080	$137,000.00
13131 Wedel Rd Trufant, MI 49347	$135,000.00
2595 W Mayo Dr Fremont, MI 49412	$133,000.00
66266 M-66 Sturgis, MI 49091	$132,900.00
11226 Oak Dr #9 Mecosta, MI 49332	$129,900.00
160 Sutherland Dr Stanton, MI 48888	$128,000.00
785 Lake Shore Pt Coldwater, MI 49036	$125,000.00
3857 Kehoe Dr NE Ada, MI 49301	$123,657.00
4627 White Rd Pierson, MI 49339	$123,000.00
10641 S Westnedge Ave Portage, MI 49002-7350	$122,500.00
350 Oliverda Dr Sherwood, MI 49089	$120,000.00
338 Carpenter Dr Battle Creek, MI 49017	$120,000.00
14909 White Creek NE Cedar Springs, MI 49319	$119,000.00
6222 Interlochen Fountain, MI 49410	$118,000.00
365 Sunset Dr Dowling, MI 49050	$114,900.00
5584 Ridge Dr Reading, MI 49274	$112,000.00
62360 Cond Dr Jones, MI 49061	$112,000.00
14556 Lonchar Dr Climax, MI 49034	$110,500.00
55290 8th Ave Grand Junction, MI 49056-8405	$110,000.00
1524-A W Chilberg Rd Ludington, MI 49431	$110,000.00
69766 Krontz Rd Sturgis, MI 49091-8421	$108,000.00
13038 Lake Breeze Sand Lake, MI 49343	$107,000.00
13941 Trout St Mecosta, MI 49332	$107,000.00
7160 E Lakeshore Dr Walkerville, MI 49459	$105,000.00
2265 Tamarack Rd Lake Odessa, MI 48849	$104,000.00
6109 W Lakeview Dr Fremont, MI 49412	$103,100.00
452 N Payne Lake Rd Middleville, MI 49333	$99,900.00
24230 Banker St Sturgis, MI 49091	$99,000.00
4415 N Gravel Ridge Rd Coral, MI 49322	$97,000.00
310 Seventh St Lakeview, MI 48850	$95,000.00
1919 Ottawa Trl Hastings, MI 49058	$94,575.00
10300 Alpine Dr Reed City, MI 49677	$92,500.00
7751 Topinabee Montgomery, MI 49255	$92,000.00
3283 Sunview Ln Arcadia, MI 49613	$91,900.00
1222 N Blossom Lake Dr Colon, MI 49040	$90,000.00
2467 Wasabinang St #1 Hastings, MI 49058	$90,000.00
10071 N Hagen Dr Irons, MI 49644	$89,900.00
12185 Black Creek Gowen, MI 49326	$85,000.00
1233 Hess Lake Dr Grant, MI 49327	$83,750.00
1615 W 17 Mile Rd Bitely, MI 49309	$83,000.00

59256 Lakeshore Dr Colon, MI 49040-9226	$82,218.00
9831 Kathleen St Grant, MI 49327	$82,000.00
724 Fountain Ln #52 Coldwater, MI 49036	$79,000.00
82 Sundago Park Hastings, MI 49058	$79,000.00
4392 Lynden Rd Shelbyville, MI 49344	$78,000.00
612 Dockside Ln Coldwater, MI 49036	$77,500.00
8871 W Elbow Irons, MI 49644	$77,000.00
513 Beechwood Ln Coldwater, MI 49036	$76,000.00
2600 Dale Dr Six Lakes, MI 48886	$75,000.00
2790 W Wilmas Way Baldwin, MI 49304	$72,500.00
10101 N Holland Rd Edmore, MI 48829	$71,500.00
5871 E Ford Lake Dr Fountain, MI 49410	$70,000.00
7780 Wildwood Rd Reading, MI 49274	$70,000.00
5428 E Deer Rd Fountain, MI 49410	$69,900.00
126 Wildwood Beach Quincy, MI 49082	$69,000.00
9207 S Trail of the Lakes Baldwin, MI 49304	$68,500.00
5383 Birch Island Dr #101 Barryton, MI 49305	$68,000.00
230 W Pickerel Lake Dr Newaygo, MI 49337	$66,250.00
5148 S Lazy Deer Ln Baldwin, MI 49304	$65,000.00
686 Buckeye Coldwater, MI 49036	$63,500.00
305 Olsen Dr Sheridan, MI 48884	$62,000.00
8390 Chain-O-Lakes Delton, MI 49046	$60,000.00
20657 N Water Way Dr Chippewa Lake, MI 49320	$60,000.00
4104 Wilson Beach Rd Twin Lake, MI 49457	$58,000.00
726 Fountain Ln Coldwater, MI 49036	$57,970.00
169 Waffle Pt Coldwater, MI 49036	$56,500.00
4150 Lakeview Dr #1 Hillsdale, MI 49242	$55,684.00
310 E Main St Hart, MI 49420	$55,000.00
7776 Island Dr Montgomery, MI 49255	$54,000.00
2569 Lake Shore Dr Niles, MI 49120	$50,000.00
1248 Channel Dr Bitely, MI 49309	$50,000.00
2320 Eleen Six Lakes, MI 48886	$49,900.00
4684 Beech Lakeview, MI 48850	$49,000.00
124 Wildwood Beach Quincy, MI 49082	$45,000.00
5974 McKibben Rd Delton, MI 49046	$44,730.00
876 W Oak St Bitely, MI 49309	$42,000.00
8374 Chain-O-Lakes Delton, MI 49046	$40,000.00
