# Supplementary material for: Understanding Acceptable Level of Risk: Incorporating the Economic Cost of Under-Managing Invasive Species
Source: PLoS One. 2015 Nov 4;10(11):e0141958. doi: 10.1371/journal.pone.0141958 (PMC4633185; doi:10.1371/journal.pone.0141958)
Supplement: S4 Supplementary Information — (DOCX) [file pone.0141958.s005.docx]

S4 Supplementary Information.

Data related to: “The cost of species introductions related to control efforts was determined by contacting twelve Michigan lake groups that fund control of aquatic macrophytes”:

| **Name** | **Budget** |
| --- | --- |
| Upper Long Lake | 98252.52 |
| Bass Lake | 24800.00 |
| Langford lake | 45000.00 |
| Barton Lake | 22000.00 |
| Elizabeth Lake | 143541.04 |
| Duck Lake | 16000.00 |
| Walled Lake | 97435.00 |
| Chippewa Lake | 114949.00 |
| Sanford Lake | 112600.00 |
| Paradise Lake | 82000.00 |
| Wixom Lake | 262350.00 |
| Lake Mitchell | 209274.48 |
